# Synaptic Plasticity in Cortical Inhibitory Neurons: What Mechanisms May Help to Balance Synaptic Weight Changes?

**DOI:** 10.3389/fncel.2020.00204

**Published:** 2020-09-04

**Authors:** Nicholas M. Bannon, Marina Chistiakova, Maxim Volgushev

**Affiliations:** Department of Psychological Sciences, University of Connecticut, Storrs, CT, United States

**Keywords:** inhibitory neurons, neocortex, hippocampus, synaptic plasticity, homeostasis, homosynaptic plasticity, heterosynaptic plasticity

## Abstract

Inhibitory neurons play a fundamental role in the normal operation of neuronal networks. Diverse types of inhibitory neurons serve vital functions in cortical networks, such as balancing excitation and taming excessive activity, organizing neuronal activity in spatial and temporal patterns, and shaping response selectivity. Serving these, and a multitude of other functions effectively requires fine-tuning of inhibition, mediated by synaptic plasticity. Plasticity of inhibitory systems can be mediated by changes at inhibitory synapses and/or by changes at excitatory synapses at inhibitory neurons. In this review, we consider that latter locus: plasticity at excitatory synapses to inhibitory neurons. Despite the fact that plasticity of excitatory synaptic transmission to interneurons has been studied in much less detail than in pyramids and other excitatory cells, an abundance of forms and mechanisms of plasticity have been observed in interneurons. Specific requirements and rules for induction, while exhibiting a broad diversity, could correlate with distinct sources of excitatory inputs and distinct types of inhibitory neurons. One common requirement for the induction of plasticity is the rise of intracellular calcium, which could be mediated by a variety of ligand-gated, voltage-dependent, and intrinsic mechanisms. The majority of the investigated forms of plasticity can be classified as Hebbian-type associative plasticity. Hebbian-type learning rules mediate adaptive changes of synaptic transmission. However, these rules also introduce intrinsic positive feedback on synaptic weight changes, making plastic synapses and learning networks prone to runaway dynamics. Because real inhibitory neurons do not express runaway dynamics, additional plasticity mechanisms that counteract imbalances introduced by Hebbian-type rules must exist. We argue that weight-dependent heterosynaptic plasticity has a number of characteristics that make it an ideal candidate mechanism to achieve homeostatic regulation of synaptic weight changes at excitatory synapses to inhibitory neurons.

## Introduction

Inhibition in cortical networks serves a multitude of functions, including balancing and restricting the spread of excitation (Wehr and Zador, 2003; Okun and Lampl, 2008; Ozeki et al., 2009; Moore et al., 2018), organizing neuronal activity in temporal and spatial patterns (Klausberger and Somogyi, 2008; Adesnik and Scanziani, 2010; Cardin, 2018; Unal et al., 2018), and shaping response selectivity of cortical neurons (Vidyasagar et al., 1996; Monier et al., 2003; Barnes et al., 2015). Adaptive fine-tuning of inhibition, necessary for achieving these functions, is mediated by synaptic plasticity. Plasticity of inhibitory systems can be mediated by changes at inhibitory synapses and also by changes at excitatory synapses at inhibitory neurons. Here, we consider that latter locus: plasticity at excitatory synapses to inhibitory neurons.

Plasticity of excitatory synaptic transmission to interneurons has been investigated in much less detail than in pyramidal neurons and other excitatory neurons. Inhibitory interneurons, while representing about 10–20% of the total number of neurons in different cortical areas, express a remarkable diversity of types, serving distinct roles in the operation of cortical networks and characterized by a distinct morphology, electrophysiology, and pattern of protein expression (Kawaguchi and Kubota, 1997; Markram et al., 2004; Ascoli et al., 2008; Battaglia et al., 2013; Druckmann et al., 2013; Jiang et al., 2015; Tremblay et al., 2016). Research has shown that excitatory inputs to these diverse types of inhibitory neurons express a multitude of forms and mechanisms of plasticity (reviewed in Bischofberger and Jonas, 2002; Galván et al., 2011; Kullmann and Lamsa, 2011; Laezza and Dingledine, 2011; Topolnik, 2012; Pelkey et al., 2017; Topolnik and Camiré, 2019), including Hebbian-type plasticity (Alle et al., 2001; Lamsa et al., 2005; Lu et al., 2007; Le Roux et al., 2013). Hebbian-type rules introduce positive feedback on synaptic weight changes: Potentiation of a synapse makes it more effective in evoking action potentials and, thus, increases the probability of further potentiation of that synapse. Similarly, depression of a synapse decreases its chances to evoke a spike and be potentiated, thus increasing the probability of its further depression. This positive feedback makes synaptic weights prone to runaway potentiation or depression and eventual saturation, which may impair the ability of synapses for further adaptive changes and compromise stability of operation of neurons and neuronal networks. However, synaptic weights in real inhibitory neurons do not express runaway dynamics and remain within an operational range, and neuronal networks of the brain operate in a regime of balanced excitation and inhibition. This implies the existence of additional plasticity mechanisms that counteract the tendency for runaway dynamics of synaptic weights introduced by the positive feedback of Hebbian-type rules. We argue that such homeostatic regulation of synaptic weight changes can be achieved by heterosynaptic plasticity at excitatory synapses to inhibitory neurons.

To appreciate the context in which candidate homeostatic mechanisms operate, we first consider diverse forms and mechanisms of plasticity of excitatory synapses at various types of inhibitory neurons. The diversity of plasticity forms in interneurons highlights the need for a generic and robust homeostatic mechanism(s). A candidate mechanism that fulfills these requirements is calcium-dependent heterosynaptic plasticity. Therefore, we next consider calcium sources that can trigger plasticity in interneurons and evidence for heterosynaptic plasticity, including a novel form of weight-dependent heterosynaptic plasticity that we have recently described for major electrophysiological types of inhibitory neurons. Finally, we discuss how these diverse forms of plasticity might affect the overall excitatory drive of inhibitory neurons, and which of these forms of plasticity could contribute to homeostatic regulation of synaptic weights of excitatory inputs to inhibitory neurons.

## Diverse Forms and Mechanisms of Plasticity of Excitatory Inputs to Inhibitory Neurons

Research into plasticity of excitatory synaptic transmission to inhibitory neurons has revealed that mechanisms of plasticity can be connection-specific, i.e., determined by the identity of both the presynaptic and postsynaptic cells. Therefore, the description of plasticity studies below is organized both historically and by specific connections, defined by the location of interneurons and the source of axons forming the synapses. Throughout the description, we accentuate two further points that are important for the purposes of this review. First, that outcome of plasticity experiments is typically not uniform, implying that, in addition to the type of connection and detail of the plasticity induction protocol, further factors are involved in determining whether the result will be long-term potentiation (LTP), long-term depression (LTD), or no change. Second is the issue of input specificity of plastic changes. Because heterosynaptic plasticity might play a central role in balancing synaptic changes, we point to evidence for heterosynaptic changes even when considering results of studies aimed at investigation of homosynaptic plasticity (see Box 1 for definitions and discussion).

Box 1 **Homosynaptic and Heterosynaptic Plasticity.**

Two main protocols are most commonly used for the induction of long-term plasticity of synaptic transmission: afferent tetanization and pairing. Either protocol can induce *homosynaptic plasticity*, changes at synapses that were activated during the induction (inputs in red with arrows denoting stimulation during the induction). Co-occurring alongside homosynaptic plasticity is *heterosynaptic plasticity*, defined as changes at inputs that were not stimulated during the induction protocol (inputs in green, marked with question marks).

A complicating factor in the concept of homosynaptic plasticity is the nature of “input-specificity.” Conventionally, plasticity is called input-specific if no changes are observed in an independent test input, not stimulated during the induction, i.e., no heterosynaptic changes. In a strict sense, input specificity means changes *only* at activated synapses and not at any other of the hundreds or thousands of synapses on the postsynaptic neuron, only a few of which were contributing to the tested heterosynaptic response. Assessing changes at all unstimulated inputs is technically intractable. At the same time, all studies that specifically addressed changes at nearby synapses found that input-specificity breaks down at short distances (Schuman and Madison, 1994; Engert and Bonhoeffer, 1997; Royer and Paré, 2003). Also at synapses distant from those stimulated during the induction, heterosynaptic plasticity is often induced, e.g., by calcium rises produced by back-propagating spikes (for further discussion see Chistiakova et al., 2014, 2015). Note that unlike input specificity, the wording “homosynaptic plasticity” makes no assumptions and has no implications about possible changes (or absence of changes) at other synapses that were not tested. Therefore, in this review, we use “homosynaptic plasticity” to refer to changes at synapses activated during the induction, and “heterosynaptic plasticity” to refer to changes at synapses that were not activated during the induction.

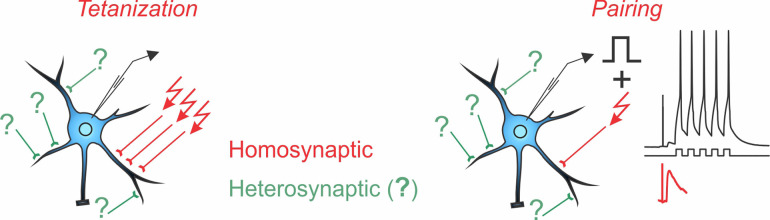

**Figure 1:**
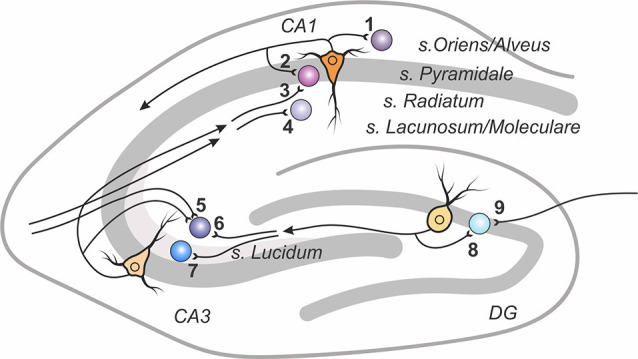
A scheme of excitatory connections to inhibitory interneurons in the hippocampus, in which synaptic plasticity was studied. Excitatory contacts onto interneurons of specified lamina in the dentate gyrus, areas CA1 and CA3 are displayed with reference number corresponding to cited research in Table 1.

The hippocampus represents a classical experimental system to study synaptic plasticity, and it has been a structure of choice for most studies of plasticity of excitatory synaptic transmission to inhibitory interneurons (Figure 1 and Table 1).

**Table 1:** Plasticity in inhibitory neurons in the hippocampus and neocortex.

Reference Cortical region (connection #)	Layer cell type	Stim site/Input	Induction protocol	Blocker/Agonist in bath	Homosynaptic LTP, LTD, No (out of *N* cases)	Heterosynaptic	Mechanisms/Involved receptors/Ca++ source/Cascades	Pre/Post (measure)	Species Age rec T°
Taube and Schwartzkroin (1987) *Hippocampus CA1* (***3***)	Pyramidale/ oriens border basket	S. radiatum	HFS: 100 stimuli @100 Hz	—	3 LTP; 3 LTD; 6 No (*n* = 12)	—	—	—	Guinea pig adult 35°C
McMahon and Kauer (1997) *Hippocampus CA1* (***4***)	Radiatum basket, bistratified	S. radiatum Schaffer collat	HFS: 100 stimuli @100 Hz, x2@0.05 Hz; Pairing: 60 stim @1 Hz +depolariz. to −10 mV	PTX	HFS: 3 LTP; 32 LTD; 14 No (*n* = 49); Pairing did not induce plasticity	Heterosynaptic LTD (8 out of 8 tested)	—	—	SD rat P16-26 29–31°C
Cowan et al. (1998) *Hippocampus CA1* (***3***)	Pyramidale FS	S. radiatum	HFS: 40 stimuli @100 Hz, x4@0.1 Hz, alone or with depolarization	Bic or PTX	HFS: 0 LTP; 6 LTD; 5 No; (*n* = 11); HFS + Dep: 10 LTP; 17 LTD; 8 No (*n* = 35)	HFS: 2 LTP; 3 LTD; 6 No; (*n* = 11); HFS + Dep: 9 LTP; 18 LTD; 8 No (*n* = 35)	Ca++ dependent, blocked by BAPTA	—	Wistar rat P17–25 29–31°C
Wang and Kelly (2001) *Hippocampus CA1* (***3***)	Pyramidale FS non-pyramidal neurons	Schaffer collaterals/ comissural fibers	Pairing: 30 stimuli at 1 Hz with depolarization to 0 mV	—	LTP (to about 200%);	—	Ca++ dependent, blocked by BAPTA; reduced ( 150% ctrl instead) by APV; CaMKII-dependent, blocked by Ca-binding peptide or autoinhibitory CaMKII(281–301) in the pipette, potentiation by activation of CaMKII occludes LTP;	—	SD rat P18–22 31°C
Perez et al. (2001) *Hippocampus CA1* (***1, 4***)	Oriens or radiatum	S. oriens or radiatum; minimal stim	TBS: 4 stimuli @100 Hz paired with 60 ms steps to −20 mV; x5@5 Hz; x3@0.033 Hz;	—	Oriens: LTP (*n* = 15); No changes if TBS alone (*n* = 8) or depolarization steps alone (*n* = 8); radiatum: No changes (*n* = 8)	—	NMDA-independent; prevented by mGluR-I/II blockers or selective mGluR1a antagonist;	S. oriens: pre (failure rate);	Rat P18–21 22–24°C
Lamsa et al. (2005) *Hippocampus CA1* (**4**)	Radiatum	S. radiatum	Pairing: 120 stimuli at 2 Hz at *V*h = 0 mV, pulses (a) or continuous (b)	PTX + CGP–52432	Pairing (a) 16 LTP, 0 LTD, 14 No (*n* = 30); Pairing (b) 17 LTP, 0 LTD, 28 No (*n* = 45)	Excluded from analyses (“LTP defined as >25% pathway-specific potentiation”)	Ca++ dependent; NMDA-dependent	Post (no PPR changes)	SD rat P21–28
Lamsa et al. (2007) *Hippocampus CA1* (***1, 2***)	Pyramidale oriens//alveus bi-stratified, axo-axonic, basket; RS or FS	Alveus	HFB: 5 stim@100 Hz x5@4-5 Hz, x4@0.1 Hz; Pairing: 100 stimuli at de- or hyperpolariz. Phase of a 4 Hz sine wave; HFS: 100 stimuli @100 Hz x2@0.1 Hz	PTX + CGP–52432	HFB, single stimuli or HFS, paired with depolarization (current injection or strong stimuli): No changes; with hyperpolarization: LTP (“anti-Hebbian”)	Not considered; though clear cases for heterosynaptic LTP in scatters	Ca++ dependent; NMDA independent; CP-AMPA dependent;	—	SD rat P21–28 31–32°C
Topolnik et al. (2006) *Hippocampus CA1* (***1***)	Oriens//alveus	S. oriens	TBS: 4 stimuli @100 Hz paired with 60 ms steps to −20 mV; x5@5 Hz; x3@0.033 Hz;	—	LTP (*n* = 5); LTP if ERK, Srk or intracellular Ca++ release alone blocked; but LTD if combinations are blocked, or TRP receptor blocked	—	Ca++ imaging; mGluR1α and mGluR5 involved in fast and slower Ca++ signals; sources of intracellular Ca++ increase; LTP induction by TBS with dep pulses: still LTP if ERK, Srk or intracellular Ca++ release alone blocked; but LTD if combinations are blocked; also, block of TRK receptors -> LTD	Pre (failure rate)	SD rat P15–23 31–33°C
Jia et al. (2010) *Hippocampus CA1* (***1***)	Oriens, nicotine-sensitive cells; PV−; some are NPY+,CR+, SST+,VIP+	S. oriens	HFS: 100 stimuli @100 Hz, VC −70 mV	APV; Bic; MLA; atropine	No (*n* = 4) in ‘control cocktail’; LTP in 10 μM (*n* = 4) or 1 μM (*n* = 5) nicotine;	—	NMDA-independent; required nicotine receptors (with the used blockers); Ca++ dependent, blocked by BAPTA, but not by ryanodine or nifedipine; nicotine induces Ca++ influx *via* activation of non α-7 AChRs, also with APs blocked;	—	SD rat P18–54; 30°C
Nissen et al. (2010) *Hippocampus CA1* (***1, 2, 3, 4***)	Pyramidale, radiatum, oriens; PV+; NPY+; SST+; CBR1+; axo-axonic, basket, bi-stratified, non-basket	S. oriens/ alveous; in some expts ctrl eld in S. radiatum	HFS: 100 stim @100 Hz x2@0.05 Hz at −70 or −90 mV; TBS: 5 stimuli @100 Hz, x4@5 Hz, x5@0.05 Hz	APV; PTX + CGP–55845	Perisomatic-targeting (*n* = 14): LTP in PV+ CB1R− (7/8); No in PV− CB1R+ (6/6); Dendrite-targeting, bistratified PV+: 0 LTP, 5 LTD, 2 NO (*n* = 7); PV− CB1R+: No (5/5); CB1R+: No plasticity (14/14) after HFS or TBS even without APV	Excluded from analyses (LTP defined as >25% pathway-specific potentiation)	CP-AMPAR involved; CP-AMPARs present in PV+ (low RI, *n* = 45 inputs) but not in CB1R+ cells (high RI > 0.5 in 25/30 inputs)	—	SD rat P21–28; 31–33°C
Szabo et al. (2012) *Hippocampus CA1* (***1, 4***)	Radiatum (ivy cells; Schaffer-Collaterals associated cells) oriens (O-LM cells)	S. radiatum (for ivy cells); S. oriens (for O-LM cells)	TBS: 5 stimuli @100 Hz, x4@4 Hz, x10@0.05 Hz; sometimes with depolarization pulses	APV; PTX + CGP–55845; AM-251	LTP in Ivy NOS+ cells (6/6) and O-LM SM+ cells (6/6); No LTP if TBS paired with depolarization (Ivy 5/5; O-LM 7/7); No LTP in SCA CCK+ CB1R+ cells (*n* = 5 TBS; *n* = 5 TBS with depolarization)	Not considered	CP-AMPARs are necessary; present in ivy and O-LM cells	Pre (CV^−2^)	SD rat P21–28; 31–33°C
Griguoli et al. (2013) *Hippocampus CA1* (***1***)	Oriens SST+ cells	S. oriens/alveus	HFS: 100 stimuli @100 Hz x2@0.1 Hz + hyperpolarization to −90 −100 mV	APV; gabazine + CGP–54656	LTP in control (*n* = 17); with α7-nAChRs blocked: 6 LTP, 11 No (*n* = 17); with α7-nAChRs, mGluR-I and mGluR1/5 blocked: No changes (*n* = 15); α7−/− mice: 1 LTP, 17 No (*n* = 18)	—	Ca++ influx through α7 nicotinic CP-AChRs is necessary for ‘anti-Hebbian’ LTP	pre	Mouse P14–21; C57BL/6 or alpha7−/−
Le Roux et al. (2013) *Hippocampus CA1* (***2, 3***)	Pyramidale PV+	S. radiatum for FF Schaffer collateral inputs; S. oriens/alveus for FB inputs	400 stimuli @5 Hz, at 0 mV for Hebbian; at −90 mV for anti-Hebbian; Also: 900 pulses @ 0.1, 1, 5, 20 Hz; and 100 stim@100 Hz x5;	Bic	Anti-Hebbian: LTP in both FF and FB inputs (*n* = 11; *n* = 9); Hebbian: LTP in FB only (*n* = 8), but No in FF (*n* = 13);	No, input-specific only	Anti-Hebbian required CP-AMPA; Hebbian required NMDA; differential frequency-dependence (BCM-curve)	Post (PPR, NASPM-block; responses to uncaged Glu)	Mouse P17–23; 31°C
Camiré and Topolnik (2014) *Hippocampus CA1* (***1***)	Oriens FS, basket and bi-stratified	S. oriens/ alveus, distal inputs	TBS: 3 stimuli @100 Hz, x8@4 Hz, x3@0.033 Hz	Gabazine + CGP–55845	Sub-threshold TBS, small amplitude Ca++ transients: LTP (*n* = 7); Supra-threshold TBS, large supralinear Ca++ signals: LTD (*n* = 7); if supralinear Ca++ summation is blocked with CPA: LTP after strong TBS	—	Ca++ signals (imaging): CP-AMPARs; less NMDA; small contribution of L-type VGCC; supralinearity of Ca++ signals produced by burst stimulation was eliminated by NASPM block of CP-AMPARs; CPA or ryanodine block of Ca-induced Ca++ release; but not by blocking NMDARs or VGCC (L,T,R)	—	Mouse P13–21; 30–33°C
Nicholson and Kullmann (2014) *Hippocampus CA1* (***1***)	Oriens regular firing	Alveus/oriens border	HFS: 100 stimuli @100 Hz x2@0.05 Hz; APs only: 500 pA 500 ms depolarization x20@0.2 Hz	APV; PTX + CGP–55845	HFS: LTP (*n* = 29); APs only: LTP (*n* = 15);	No, input-specific after HFS (yes, after AP only)	Ca++ dependent (blocked by 25 mM BAPTA); no involvement of NO; no involvement of TRPV1; occlusion between HFS-induced and AP-only induced LTP	HFS: pre (failure rate, PPR, spont freq) APs only: pre (PPR, spont freq)	Mouse P21–25
Nicholson and Kullmann (2017) *Hippocampus CA1* (***1***)	Oriens regular firing	Alveus/oriens border	HFS: 100 stimuli @100 Hz x2@0.05 Hz; APs only: 500 pA 500 ms depolarization x20@0.4 Hz	APV; PTX + CGP–55845	HFS and APs-only induced LTP	No, input-specific after HFS (yes, after AP only)	T-type Ca++ channels contribute to both HFS-induced and APs-only induced LTP	—	Mouse P16–23
Maccaferri et al. (1998) *Hippocampus CA3* (***7***)	Lucidum or border to radiatum	DG mossy fibers	Tetanic stimulation of MF, parameters not specified	Bic; APV	0 LTP; 6 LTD; 3 No (*n* = 9)	—	NMDA-independent; Ca++ independent, NOT occluded by forskolin	Presyn (failure rate)	SD rat; P14–20; 24°C
Laezza et al. (1999) *Hippocampus CA3* (***5***)	Radiatum	CA3 pyramid. layer; continuum of CP-AMPAR–– CI-AMPAR synapses	HFS: 30 stimili @100 Hz, x3@0.1 Hz	Bic; APV	CP-AMPAR synapses: LTD (12/12); CI-AMPAR synapses: 7 LTP; 3 No (*n* = 10)	—	LTD at CP-AMPAR synapses: Ca++ dependent, abolished by 30 mM BAPTA or clamp at +40 mV; mGluR7-dependent, prevented by group II/III mGluR antagonist LY341495 (without affecting basal transmission)	Presyn (failure rate)	SD rat, P10–16
Laezza and Dingledine (2004) *Hippocampus CA3* (***5***)	Radiatum	CA3 pyramidal layer; continuum of CP-AMPAR–– CI-AMPAR synapses; No correlation with NMDA-component	HFS: 30 stimili @100 Hz, x3@0.1 Hz; Pairing: 120 stim @1 Hz, at −25 mV;	Bic	CP-AMPARs: LTP after HFS at −30 mV (*n* = 6) or Pairing at −25 mV (*n* = 4); HFS at 0 mV 1 LTP; 4 LTD; 1 No (*n* = 6); HFS at −70 mV 1 LTP; 4 LTD; 2 No (*n* = 7); LTD after HFS at −30 mV with 30 mM BAPTA (*n* = 4); CI-AMPARs: No changes after HFS at −30 mV (*n* = 6);	—	CP-AMPAR with NMDAR synapses: both LTP and LTD were NMDAR-dependent; but with intracellular BAPTA HFS induced LTD; CP-AMPAR synapses lacking NMDAR: LTD was induced by pairing	—	SD rat P9–12; RT
Lei and McBain (2002) *Hippocampus CA3* (***7***)	Lucidum	DG mossy fibers; continuum of synapses: CP-AMPAR with low NMDA/AMPA — CI-AMPAR with high NMDA/AMPA	HFS: 100 stimili @100 Hz, x3@0.1 Hz	Bic; Glycine	LTD at both CI and CP-AMPAR synapses (*n* = 5; *n* = 6); with APV: No changes at CI-AMPAR synapses (*n* = 6); LTD at CP-AMPAR synapses (*n* = 7)	—	Ca++ dependent (blocked with 20 μM BAPTA); NMDAR-dependent in CI-AMPAR synapses; NMDAR-independent in CP-AMPAR synapses	—	SD rat; P16–20
Lei and McBain (2004) *Hippocampus CA3* (***7***)	Lucidum	DG mossy fibers	HFS: 100 stimili @100 Hz, x3@0.1 Hz	Bic	LTD at both CI- and CP-AMPAR synapses	—	CI-AMPAR syn: NMDA-dependent; AMPA-trafficking; CP-AMPAR syn: NMDA-independent;	CI-AMPAR syn: post; CP-AMPAR syn: pre; (CV, PPR, NMDA-resp, use-depend AMPAR-block)	SD rat; P16–20; 22–24°C
Pelkey et al. (2005) *Hippocampus CA3* (***7***)	Lucidum	DG mossy fibers	HFS: 100 Hz 1 s, x3@0.1 Hz	Bic; APV	LTD in control (*n* = 10); after reduction by mGluR7 agonist AP4 responses recover to control after HFS (is it “LTP” ?)	No; no changes at synapses from CA3 collaterals	LTD blocked by mGluR7-antagonist MCOG; PKC-dependent. APV all times in the bath -> only NMDA-independent component	Presyn (PPR, CV, failure rate)	Mouse C57BL/6 P12–22; 22–25°C, some at 33–35°C

Galván et al. (2008) *Hippocampus CA3* (***6***)	Lacunosum/ moleculare	DG mossy fibers; 18/28 CI-AMPARs; 10/28 CP-AMPARs	HFS: 100 stimuli @100 Hz **+ depolarization**, x3@0.1 Hz	Bic; APV	With CP-AMPARs blocked by PhTx: synapses with mostly CI-AMPARs showed associative LTP (*n* = 11, inp-specific, no in C-A inputs); synapses with initially stronger but blocked CP-AMPAR component showed No changes (5/7) or LTD (2/7) of the remaining CI-AMPAR mediated component; Without Ph-Tx: 25 LTP; 2 LTD; 5 No (*n* = 32)	No; no changes at comiss/ associate synapses from CA3 in experiments with PhTx	Ca++ dependent. Prevented by hyperpolarization (*n* = 10), L-type VGCC (*n* = 9); by 20 mM BAPTA in 11/15 cells. NMDA-R independent; requires mGluR1, IP3 and RyR; with mGluR1, IP3 blocked or RyR-release depleted LTD was induced.	Both LTP and LTD: pre; (PPR, CV, failure rate)	SD rat, P22+4; 33 ±1 °C;
Galván et al. (2015) *Hippocampus CA3* (***6***)	Radiatum or lacunosum/ moleculare	DG mossy fibers	HFS: 100 stimuli @100 Hz + **depolarization**, x3@0.1 Hz	—	in Bic + APV: LTP (*n* = 6)	No; no changes in RC inputs in experiments with blockers of CaMKII or PKC	LTP in MF: NMDA-independent; not blocked by CaMKII inhibitors KN62 or KN-93; requires postsynaptic PKC: LTD with intracellular PKC blocker chelerythrine (*n* = 9);		SD rat, P35+5; 33 ± 1°C
Galván et al. (2015) *Hippocampus CA3* (***5***)	Radiatum or lacunosum/ moleculare	CA3 pyramid. layer, recurr collaterals; 19/26 CI− 5/26 CP–AMPAR syn; (2:atypical IV)	HFS: 100 stimuli @100 Hz + **depolarization**, x3@0.1 Hz	—	in Bic + APV: CP-AMPARs: LTD (5/5); CI-AMPARs: transient potentiation (10/19), No (9/19); Bic (no APV): LTP at CI-AMPAR (*n* = 8);	No; no changes in MF inputs in experiments with blockers of CaMKII or intracellular PKC	LTP in RC CI-AMPARs was blocked by: hyperpolarization to −100 mV; APV; intracellular 20 mM BAPTA; bath application of CaMKII inhib KN-62 or KN-93; intracellular PKC blocker chelerythrine; not blocked: by mGluR1 or mGluR5 blockers	Presyn (PPR, CV)	SD rat, P35+5; 33±1°C
Pan et al. (2019) *Hippocampus CA3* (***7***)	Lucidum	DG mossy fibers; only CP-AMPARs included, with Rectification Index <0.3	HFS (not specified)	PTX; APV	WT mice (controls, *n* = 84): 0 LTP, 70 LTD, 14 No; TrkB −/− or blocked: 5 LTP, 4 LTD, 8 No (*n* = 17); TrkB/PLC signaling blocked: 6 LTP, 8 LTD, 11 No (*n* = 25); BDNF −/− or scavenged: 3 LTD, 10 No (*n* = 13); CB1R antagonists: 4 LTP, 0 LTD, 14 No (*n* = 18); CB1R−/−: 3LTP, 2LTD, 1No	—	LTD at CP-AMPARs: NMDA-independent; prevented or converted to a mix LTP/LTD/No, if BDNF/TrkB/PLC signaling is blocked or impaired; or CB1Rs are blocked or deleted.	Presyn (PPR)	Mouse WT or conditnl TrkB−/− BDNF−/− CB1R−/− P21–29; Room T°
Alle et al. (2001) *Hippocampus* *Dentate gyrus* (***8***)	DG basket cells	DG mossy fibers; connected pairs granule-basket or extracell stim	HFS: 25stim@30 Hz, x12@0.33 Hz, x3@0.011; associative HFS: + BS spikes by depolariz. Pulses; nonassociative HFS: BS at −70 mV	—	LTP after associative aHFS; LTD after non-associative nHFS	—	LTP attenuated by 30 mM BAPTA (not by 10 mM) and reduced by by PKC-antagonist bisindolylmaleimide; not by PKA blocker H-89	Both LTP and LTD: presyn, (failure rate, CV, PPR)	Wistar rat P18–25; 34 ± 2°C
Sambandan et al. (2010) *Hippocampus* *Dentate gyrus* (***8, 9***)	FS perisomatic inhibitory neurons (PII)	DG mossy fibers (MF): CP-AMPARs and NMDAR; perforant path from enthorhinal ctx (PP): CI-AMPARs and low levels of NMDARs	BFS: 25 stim@30 Hz, x12@0.33 Hz, x3@0.033 Hz; associative BFS: PP (*dt* = 10 ms) + MF, induced AP bursts; “nonassociative”: PP or MF + APs induced with 0–2 ms delay by depolariz. (*note: slower* *kinetics of PP* *may require* *different timing*)	Bic or SR95531	Associative BFS: LTP in MF, No changes in PP with 10 ms delay PP-MF; No LTP with <5 ms or >15 ms delay (*n* = 11); LTP at MF after pairing MF + depolarization-induced spikes at about 0 ms lag; No LTP at PP	No	LTP at MF synapses: NMDA-independent, requires CP-AMPA; depends on spike timing, few-ms window for induction by MF+PP pairing	—	Wistar rat P17–24; 30–34°C
Hainmüller et al. (2014) *Hippocampus* *Dentate gyrus* (***8***)	FS basket perisomatic inhibitory neurons (PII)	DG mossy fibers; connected pairs granule-basket or extracell stim	BFS: 25 stim@30 Hz, x12@0.33 Hz; associative BFS: + BS spikes by depolariz. Pulses with 1–3 ms delay; nonassociative BFS: same BFS but PII held at VC −70 mV	—	LTP after associative aBFS; LTD after nonassociative nBFS; LTP and LTD were independent, can be induced one after the other	—	Ca++ dependent; blocked by BAPTA but not by EGTA; Major Ca++ source during bursts is CP-AMPARs then NMDARs, while mGluRs, VDCCs or Ca++ stores contribute less; notably, Ca-response to single APs was not much affected by any of these; mGluR1/5 supported LTP but prevented LTD (*via* PKC activation); switch enabling MF-LTP	—	Wistar rat P17–23; 30–34°C
Lu et al. (2007) *somatosensory* *cortex*	L2/3 FS, LTS;	L2/3 pyramids; NMDAR-component about 3× stronger in PC-LTS than in PC-FS synapses	STDP 5 pre + post APs at 20 Hz, x12@0.2 Hz; pre-post delays: ±8 and ±25 ms; tested up to ±100 ms	—	LTS: LTP at +8 ms (*n* = 21); LTD at −8 ms (*n* = 12); No at +25, −25 ms (*n* = 5, 5); FS: LTD at +8 ms or −8 ms (*n* = 22, 19); No at +25 or −25 ms (*n* = 6, 8)	—	LTS: both LTP and LTD NMDAR-dependent; not sensitive to mGluR blockade by MCPG; FS: LTD did not require NMDAR; but prevented by MCPG	LTS: presyn; FS: postsyn; (CV, PPR)	SD rat, P13–16; 32–34°C
Chen et al. (2009) *somatosensory* *cortex*	L2-L4 non-FS SST+ PV−	L2-L4; 5–10 mV EPSPs; NMDA + AMPA components	TBS: 5 stimuli @100 Hz, x20@5 Hz, x6-10 times @0.1 Hz	PTX	LTP (*n* = 9); only STP, no LTP after 2–3 TBS episodes (*n* = 9); no LTP after HFS 100 Hz 1s x3 (*n* = 6);	—	NMDA-independent (*n* = 12); Ca++ independent, not blocked by 30 mM BAPTA + nimodipine; nor by *Vh* = −90 mV during TBS; blocked by incubation in PKA-inhibitors	Presyn (PPR)	Mouse P15–45, median P21 Room T°
Sarihi et al. (2008) *visual cortex*	L2/3 FS mostly PV+ basket; nonFS bitufted or bipolar	L4, half-maximal EPSPs	TBS: 4 stimuli @100 Hz, x10@5 Hz, x3@0.1 Hz + depolariz. to 0 mV ;	—	FS: 14 LTP, 0 LTD, 5No (*n* = 19); less LTP after TBS at −70 mV (6/17 cells); no LTP after depolarization alone (0/8 cells); non-FS: 6 LTP, 0 LTD, 11 No (*n* = 17);	—	FS: LTP is Ca++ dependent (blocked by 10 mM BAPTA, *n* = 8); but did not depend on NMDAR (APV, *n* = 8) or L,T type VGCC (nimodipine, Ni++, mibefradil; *n* = 11, 9, 9); required mGluR5 but not mGluR1; required PLC-IP3 system and release from internal Ca++ stores; after eye opening (P12–15) LTP did not depend on age	FS: postsyn (PPR, CV)	Mouse P12–43, most P16–19 29–31°C
Lefort et al. (2013) *visual cortex*, *(monocular V1)*	L4 FS	Connected pairs star pyramids -> FS (mostly FS->SP; occasional reciprocal SP->FS)	HFS: 10 spikes @50 Hz, x20@0.1 Hz in FS + subthreshold depolarization with 1–2 occasional spikes in SP; *note* that FS is postsyn, so mostly postsyn (FS) spiking	—	P16–17 no net changes in FS (119 ± 7.22%, *n* = 6); P22–23 net potentiation in FS (185 ± 45%, *n* = 7), reduced but not completely blocked by GABAB blocker CGP52432 (about 145%, from figure, *n* = 6);	*Heterosynaptic induction*? changes after postsynaptic FS firing, with only occasional spikes in presynaptic SP	Reduced but not completely blocked by GABAB blocker CGP52432;	Postsyn (CV)	Rat P15–23 35°C
Chistiakova et al. (2019) *visual cortex*	L1-5 FS; non-FS; diverse morpho-logical types;	Two bipolar electrodes near recording site	Pairing: synaptic stimuli to one input followed (10 ms) by 5 APs @100 Hz, x10@1 Hz, x3@0.017 Hz; Intracellular tetanization (IT): 5 APs @100 Hz, x10@1 Hz, x3@0.017 Hz, without synaptic stimuli	—	Pairing: 5 LTP, 2 LTD, 3 No (*n* = 10; net LTP); LTP in both FS and non-FS cells	Pairing, un-paired inputs: 3 LTP, 1 LTD, 6 No (*n* = 10) (No net change); IT, FS: 45 LTP, 48 LTD, 49 No (*n* = 142, No net change); IT, non-FS: 31 LTP, 10 LTD, 25 No (*n* = 66, net LTP)	Weight-dependent heterosynaptic plasticity (amplitude change correlated with initial PPR)	Presyn (PPR, CV)	Wistar rat P15–34 28–32°C
Huang et al. (2013) *visual*, *somatosensory cortex*	L2/3 FS PV+ non-FS SOM+	L4, below recording site, two bipolar electrodes	Pairing: synaptic stimuli before or after postsynaptic bursts 4 APs @100 Hz, x200@1 Hz; pre-then-post in one input, post-then-pre in the other input to the same cell; pre-post intervals: ±10, 25, 50 ms;	—	FS PV+: No changes in ctrl solution (95 ± 13%, *n* = 10 pre-then-post; 91 ± 6% *n* = 14 post-then-pre); LTD with α1-adrenoreceptors activated (methoxamine; 59 ± 5%, *n* = 6 and 51 ± 6%, *n* = 8 pre->post; 67 ± 7% *n* = 11 post->pre; 96 ± 4% *n* = 10 in un-paired); LTP with β-adrenoreceptors activated (isoproterinol; 132 ± 15%, *n* = 11 and 150 ± 25%, *n* = 9 pre->post; 130 ± 14% *n* = 12 post->pre; 102 ± 9% *n* = 14 in un-paired); STDP with both α1 and β agonists iso+met (136 ± 12% *n* = 11 pre->post and 72 ± 6% *n* = 11 post->pre at 10 ms; less at 25 ms, no at 50 ms); non-FS SOM+: similar, STDP in iso+met	No changes in un-paired, though 102±9% (SEM) *n* = 14 and 96±4% *n* = 10 might have included some LTP and LTD in individual experiments	FS PV+: Ca++ dependent, STDP in iso+met prevented by 10 mM BAPTA; NMDA-independent, APV did not prevent LTD in met, nor LTP in iso, nor STDP in iso+met; mGluR5 blocker MPEP prevented STDP in iso+met; preventing phosphorilation/trafficking of GluA1 prevented LTD, and both pre-post and post-pre pairing induced LTP; non-FS SOM+: NMDA-dependent, STDP in iso+met is prevented by APV	Postsyn (PPR)	Mouse P21–25
Kerkhofs et al. (2018) *medial prefrontal* *cortex*	L5 FS;	Bipolar electrode around dendrites	TBS: 5 stim @100 Hz x10, x3	—	Control solution: 7 LTP, 0 LTD, 3 No (*n* = 10); with adenosine A2R blocked by SCH58261: 0 LTP, 7 LTD, 3 No (*n* = 10)	—	Adenosine A2R availability controls the direction of plasticity, LTP/LTD	—	Wistar rat P35–46 32°C

*Studies of synaptic plasticity in cortical neurons are sorted by the cortical region in which inhibitory neurons were recorded and origin of presynaptic fibers. Connection # corresponds to Figure 1*.

### Plasticity of Excitatory Inputs to CA1 Interneurons in the Hippocampus

#### Diverse Forms of Calcium-Dependent Plasticity in CA1 Interneurons

The first intracellular study of plasticity of excitatory transmission to inhibitory neurons in the hippocampus aimed to reveal whether changes in excitability of interneurons could contribute to regular tetanus-induced LTP of field potentials (Taube and Schwartzkroin, 1987). Afferent tetanization did not change excitability of basket cells recorded at the border of str. pyramidale and oriens of the CA1; however, it induced plasticity of subthreshold EPSPs.

Induction of plasticity in fast-spiking (FS) neurons from CA1 str. pyramidale required [Ca^2+^]_i_ rises. Plastic changes induced by either high-frequency tetanization combined with depolarization of the recorded interneuron (Cowan et al., 1998) or pairing low-frequency stimulation with depolarization (Wang and Kelly, 2001) were prevented by adding BAPTA to the intracellular pipette solution. Blockade of NMDARs with APV did not prevent the pairing-induced LTP but reduced its magnitude (Wang and Kelly, 2001). This suggests that, although NMDARs contribute to calcium entry in FS str. pyramidale interneurons, their involvement is not critical, and rises of [Ca^2+^]_i_ necessary for triggering LTP could be achieved by engaging sources other than NMDARs. In CA1 str. oriens interneurons, induction of LTP by theta-burst stimulation paired with postsynaptic depolarization was not affected at all by NMDAR blockade but prevented by blockers of group I/II metabotropic glutamate receptors (mGluR) or selective mGluR1a antagonists (Perez et al., 2001). These neurons express calcium-permeable AMPA receptors (CP-AMPARs), which might have contributed to the Ca^2+^ influx needed for triggering LTP. Notably, the ability of the above theta-burst protocol to induce plasticity was “connection-specific”—it was effective in the str. oriens interneurons but did not induce plasticity in str. radiatum interneurons (Perez et al., 2001).

A characteristic morphological feature of inhibitory neurons is aspiny or sparsely spiny dendrites. Because, in excitatory neurons, spines are considered as the morphological substrate for restricting the spread of synaptically induced [Ca^2+^]_i_ rises, thus restricting plasticity to the activated synapses, it was proposed that, in aspiny dendrites, input-specificity of plastic changes might be compromised. Indeed, direct tests revealed a lack of input specificity of synaptic changes in CA1 inhibitory neurons (McMahon and Kauer, 1997; Cowan et al., 1998). In basket and bi-stratified neurons from str. radiatum, high-frequency tetanization induced predominantly LTD, which was not restricted to the tetanized input but could spread to nonstimulated synapses (McMahon and Kauer, 1997). In FS neurons from str. pyramidale, high-frequency tetanization combined with depolarization could induce LTP, LTD, or lead to no changes in both tetanized and nontetanized pathways in all possible combinations (Cowan et al., 1998).

Thus, initial studies demonstrate that CA1 interneurons can express long-term synaptic plasticity, which is calcium-dependent (Cowan et al., 1998; Wang and Kelly, 2001) but not input-specific (McMahon and Kauer, 1997; Cowan et al., 1998). Later research employing Ca^2+^ imaging has demonstrated that synaptically induced rises of [Ca^2+^]_i_ in aspiny dendrites do not spread much but are kept local by interneuron-specific mechanisms (Goldberg et al., 2003a; see section on calcium sources below for detail). Although the original rationale for a lack of input-specificity in interneurons because of lacking compartmentalization of calcium signals in their aspiny dendrites appeared not to be correct, experimental results demonstrating heterosynaptic plasticity (plasticity at unstimulated inputs) in inhibitory neurons remain valid. The issue of input specificity of plastic changes is further discussed in Box 1 and below in the sections on calcium signals and heterosynaptic plasticity.

These studies also found high within-experiment heterogeneity of the outcomes of plasticity induction in interneurons. Cowan et al. (1998) report, for tetanized (homosynaptic) inputs, LTP in 10, LTD in 17, and no changes in eight experiments. At nontetanized (heterosynaptic) sites, the proportion was similar: nine inputs expressed LTP, 18 LTD, and eight did not change. McMahon and Kauer (1997) observed LTD in 32 out of 49 tested inputs, LTP in three and no changes in the remaining 14. Taube and Schwartzkroin (1987) report that, out of 12 basket cells tested, three expressed potentiation, three expressed depression, and in the remaining six cells, no change or a small increase of EPSP amplitude was observed. Thus, at the same type of connection, the same induction protocol could lead to different outcomes, including induction of plasticity of the opposing sign. This suggests that additional factors, either related to cell intrinsic predispositions of synapses for plasticity or heterogeneity among stimulated input fibers or diverse subclasses of recorded neurons contribute to determining the outcome of plasticity in interneurons.

These initial studies in CA1 inhibitory neurons also demonstrate that plasticity rules and mechanisms in interneurons can be: (a) different from those known for pyramidal cells, e.g., NMDA-independent (Perez et al., 2001; Wang and Kelly, 2001); and (b) different between the different types of inhibitory neurons (Perez et al., 2001). In fact, a difference in experimental conditions as well as cell-type specificity of plasticity mechanisms and heterogeneity of recorded subpopulations of interneurons could have contributed to discrepancies between the findings of the above studies, e.g., whether CA1 inhibitory neurons express predominantly LTD (McMahon and Kauer, 1997) or also LTP (Taube and Schwartzkroin, 1987; Cowan et al., 1998) or whether pairing weak synaptic stimuli with depolarization can induce plasticity (Perez et al., 2001; Wang and Kelly, 2001) or not (McMahon and Kauer, 1997).

#### Cell and Connection-Type Specificity of Plasticity at CA1 Interneurons

Indeed, further research revealed remarkable differences in the requirements for induction and mechanisms of plasticity in different types of interneurons. At Schaffer collaterals/commissural inputs to CA1 str. radiatum interneurons, pairing synaptic stimulation with depolarization induced LTP, which required an NMDAR-mediated [Ca^2+^]_i_ rise and was expressed postsynaptically (Lamsa et al., 2005). The LTP occurred in about half of studied neurons. In contrast, in excitatory inputs from collaterals of CA1 axons to str. oriens/alveus interneurons, neither pairing synaptic stimuli with depolarization nor high-frequency bursts of strong stimuli induced LTP (Lamsa et al., 2007). LTP in these cells could be induced by high-frequency burst stimulation only if the stimuli were weak or paired with hyperpolarization of the postsynaptic cell (Lamsa et al., 2007). Induction of LTP required a [Ca^2+^]_i_ rise *via* CP-AMPARs and was not prevented by blockade of NMDARs. Requirement of this form of plasticity for hyperpolarization during the induction is explained by the increase of calcium influx *via* CP-AMPA receptors at hyperpolarized potentials, which can then reach the threshold for triggering plasticity. Because of the opposite-to-Hebbian requirement for the induction (hyperpolarization instead of depolarization and firing), this form of plasticity was called “anti-Hebbian” (Lamsa et al., 2007).

Testing CP-AMPAR-dependent plasticity in other classes of CA1 inhibitory neurons with diverse location (str. pyramidale, radiatum, oriens), morphology (axo-axonic, basket, bi-stratified, ivy, Schaffer collateral-associated cells), and pattern of expression of characteristic proteins (parvalbumin PV, neuropeptide Y, somatostatin SST, cannabinoid receptors of type 1 CBR1, nitric oxide synthase NOS) revealed further diversity of plasticity rules in interneurons (Nissen et al., 2010; Szabo et al., 2012). With NMDA receptors blocked, high-frequency stimulation paired with hyperpolarization induced LTP in perisoma-targeting PV-positive cells and LTD in dendrite-targeting PV-positive cells (Nissen et al., 2010). In NOS-positive ivy cells and SST-positive bi-stratified oriens-lacunisum/moleculare (O-LM) neurons, LTP could be induced by theta-burst stimulation, but LTP was prevented if theta-burst stimulation was paired with depolarization (Szabo et al., 2012). No CP-AMPAR-dependent plasticity could be induced in Schaffer-collateral-associated cells or in CBR1-positive neurons by any of the above protocols. Notably, CBR1-positive neurons did not express plasticity even with unblocked NMDA receptors (Nissen et al., 2010).

Different plasticity mechanisms also may be associated with different network roles of inhibitory neurons. As described above, Schaffer collateral inputs to str. radiatum interneurons, which mediate feed-forward inhibition, express Hebbian-type, NMDAR-dependent LTP (Lamsa et al., 2005). Synapses made by collaterals of CA1 pyramidal neurons onto str. oriens/alveus interneurons mediating feedback inhibition express “anti-Hebbian” LTP, dependent on calcium influx *via* CP-AMPARs (Lamsa et al., 2007). At these feedback synapses, Hebbian-type LTP could still be induced by pairing theta-burst stimulation with depolarization, but to achieve the needed [Ca^2+^]_i_ rise, activation of mGluR1α was required (Topolnik et al., 2006). Moreover, plasticity rules may be different at excitatory inputs that engage the same interneurons in either feed-forward or feedback inhibition. PV-positive interneurons in CA1 str. pyramidale receive feed-forward inputs from Schaffer collaterals and the perforant path as well as local feedback inputs from axon collaterals of local pyramidal neurons. In the feedback inputs, either Hebbian-type NMDAR-dependent or “anti-Hebbian” CP-AMPAR-dependent LTP could be induced, depending on whether synaptic stimulation was paired with depolarization (0 mV) or hyperpolarization (−90 mV). In contrast, only “anti-Hebbian” CP-AMPAR-dependent LTP could be induced at the feed-forward inputs (Le Roux et al., 2013).

Thus, the rules of induction and mechanisms of plasticity can be interneuron-type specific and even connection-type specific. In this context, “connection type” is defined by the identity of both presynaptic fibers and postsynaptic cells. Most illustrative here is the link between diverse plasticity rules and the diversity of sources of [Ca^2+^]_i_ rise, determined by the pattern of expression and subunit composition of NMDA, AMPA, and metabotropic glutamate receptors (see Box 2), which, in turn, correlates with the type of interneuron and connection.

Box 2 **Glutamate receptor channels as sources of calcium influx.**

A schematic representation of excitatory glutamatergic synapses in inhibitory and excitatory neurons and a schematic plot of current-voltage relationships of glutamate-gated ionotropic channels.

Synapses at inhibitory neurons can express NMDA receptor channels (NMDAR), calcium-permeable AMPA-receptor channels (CP-AMPAR), and calcium-impermeable AMPA receptor channels (CI-AMPAR). The proportion of CP/CI AMPARs and of AMPARs/NMDARs varies across synapses (Laezza et al., 1999; Lei and McBain, 2002; Galván et al., 2008; Lalanne et al., 2016, 2018). At mossy-fiber synapses onto CA3 str. lucidum interneurons (Lei and McBain, 2002), expression of NMDARs is inversely related to the expression of CP-AMPARs, such that synapses with more NMDARs have less CP-AMPARs (left scheme), and vice versa, synapses with less NMDARs have more CP-AMPARs (middle). At other connections, no such correlation was found. In inhibitory neurons, fast kinetics of CP-AMPARs (Geiger et al., 1995; Jonas and Burnashev, 1995; Angulo et al., 1997), a small diameter of dendrites and high buffering capacity (blue circles) help to restrict the spread of the intracellular calcium.

Glutamatergic synapses at excitatory neurons are typically formed at dendritic spines (right scheme). At spine synapses, the canonical source of calcium entry that can trigger synaptic plasticity is through NMDARs, and the spike neck helps to restrict the spread of intracellular calcium.

Synaptic current and influx of calcium through the NMDARs and CP-AMPARs have distinct dependence on voltage (rightmost plot). Owing to a magnesium block at hyperpolarized potentials, opening of NMDAR channels requires both binding of glutamate to the receptor and depolarization to relieve the pore from magnesium block. This combination of requirements makes NMDAR a “coincidence detector” for coordinated presynaptic activity that supplies glutamate and postsynaptic activation that depolarizes the dendrite. CP-AMPARs are blocked at depolarized potentials by intracellular polyamines (Rozov and Burnashev, 1999). The polyamine block leads to a characteristic rectification of the current-voltage relationship of CP-AMPARs. Rectification of the voltage-current relationship of CP-AMPARs and magnesium block of NMDARs proved to be useful for a quick electrophysiological assessment of presence and relative contribution of CP-AMPARs and NMDARs to synaptic responses (e.g., Laezza et al., 1999; Lei and McBain, 2002; Galván et al., 2008).

Calcium permeability of fast AMPAR channels depends on their subunit composition, specifically, on the presence or absence of GluRB (GluR2) subunit. AMPARs at synapses in excitatory neurons contain an edited GluRB subunit with positively charged arginine at a particular position of the pore-forming segment, which prevents calcium ions from passing through the pore. Inhibitory neurons, however, can express AMPARs that lack a GluRB subunit and are permeable for calcium. Because AMPARs are heteromers, the ratio of CP to CI AMPARs depends on the level of expression of the edited GluRB subunit (Jonas et al., 1994; Geiger et al., 1995; Jonas and Burnashev, 1995; Koh et al., 1995; Angulo et al., 1997). Relative calcium permeability of AMPARs, characterized by the ratio of P(calcium)/P(monovalent ions) varies in different types of interneurons, e.g., 1.6 in dentate gyrus basket cells; 1.4 in dentate gyrus hillar neurons; 0.7 in inhibitory neurons from layer 4 of neocortex. For comparison, this ratio is <0.1 in excitatory neurons, such as L5 pyramids from neocortex; CA3 pyramids or granule cells from dentate gyrus (Geiger et al., 1995; Jonas and Burnashev, 1995); for NMDARs the ratio is >2.5 (Koh et al., 1995; Spruston et al., 1995).

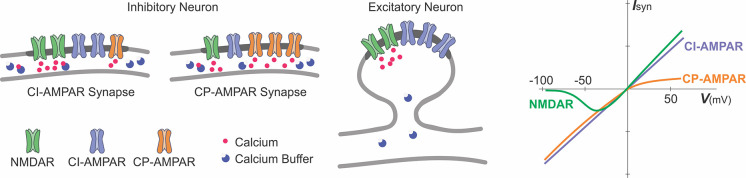

Note that specificity of plasticity rules and mechanisms with respect to the type of interneuron and connection is not strict and precise. Observed variability could be due to intrinsic variability of synapses (e.g., of the ratios of NMDA/AMPA receptors and calcium permeable/impermeable AMPA receptors across synapses within the same type of connection) and also to differences in experimental conditions and plasticity induction protocols (see Box 3 and Table 1) as well as animal lines and age and possible biases in sampling of highly heterogeneous inhibitory neurons. Regardless, an overall conclusion that plasticity in different types of interneurons and connections is mediated by different sets of mechanisms remains valid.

Box 3 **Stimulation protocols that induce plasticity in inhibitory neurons.**

Representative stimulation protocols used to induce long-term plasticity in inhibitory neurons. Note that these protocols are same or similar to those used to induce plasticity in excitatory neurons (see, e.g., Chistiakova and Volgushev, 2009).

The “single burst or train” column in the middle shows timing of individual stimuli in afferent tetanization protocols or postsynaptic potentials and postsynaptic spikes in pairing protocols. “Pattern of stimulation” shows, at a compressed scale, timing of the whole protocol, each vertical bar representing a burst or a train from the middle column. Protocols are sorted by the total number of afferent stimuli or postsynaptic spikes for pairing protocols. Any of these protocols could be used either without injection of current into the recorded neuron, or in combination with depolarizing or hyperpolarizing current. Details of the protocols used in specific studies are given in Table 1.

Afferent tetanization protocols, theta-burst stimulation (TBS), and high frequency stimulation (HFS) typically use strong stimuli that evoke spikes in the postsynaptic neuron.

Theta-burst stimulation (TBS; a; from Perez et al., 2001) consists of short, high-frequency bursts of afferent stimuli (four stimuli at 100 Hz) repeated at 5 Hz. In the illustrated example, such trains are repeated three times.

High-frequency stimulation (HFS; b–d). A canonical form of HFS uses 100 stimuli at 100 Hz (b; from Taube and Schwartzkroin, 1987). Such 1 s trains can be repeated 2–4 times every 10 s. Shorter trains (c; from Cowan et al., 1998), or stimulation at lower frequency (30 Hz, as in d; from Alle et al., 2001) are also common.

Pairing protocols (e–g) consist of pairing subthreshold stimulation with postsynaptic spikes with specific, strictly defined relative timing to induce spike-timing dependent plasticity (STDP). Bursts of postsynaptic spikes, which are more effective at causing calcium influx into the cell, are typically used. In bursts of lower frequency, each presynaptic stimulus is paired with one postsynaptic spike (20 Hz, as in e; from Lu et al., 2007). Alternatively, each presynaptic stimulus can be paired with a high-frequency burst of postsynaptic spikes (100 Hz, as in f; from Chistiakova et al., 2019, and g; from Huang et al., 2013).

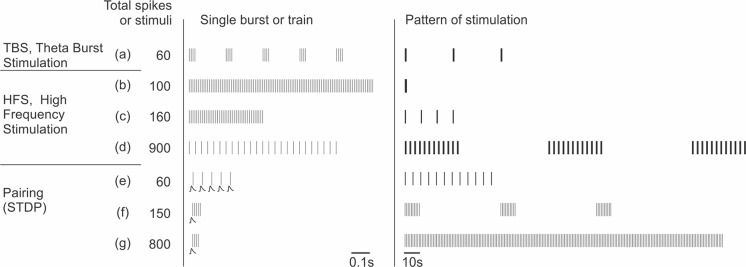

### Plasticity of Excitatory Inputs to Interneurons in the CA3 Area of the Hippocampus and Dentate Gyrus

A unique experimental model to study cell-type and connection-type specificity of plasticity of excitatory inputs to interneurons is offered by the circuitry in the dentate gyrus and CA3 region of the hippocampus (Figure 1). Axons of dentate gyrus granular cells (mossy fibers) innervate, in addition to CA3 pyramidal neurons, inhibitory neurons in CA3 and in the dentate gyrus. Inhibitory neurons in the CA3 also receive excitatory inputs from recurrent collaterals of local CA3 pyramids and commissural fibers. Interneurons in the dentate gyrus also receive input from perforant path fibers originating in the entorhinal cortex. Thus, there are different types of interneurons innervated by the same mossy fibers as well as interneurons of the same type receiving inputs from clearly distinct sources.

#### Diverse Ca^2+^ Sources Contribute to Heterogeneity of Plasticity Rules and Mechanisms in CA3 Interneurons

An initial study found that tetanic stimulation of mossy fiber inputs to CA3 interneurons induced LTD in six out of nine cells (Maccaferri et al., 1998). Like canonical presynaptic LTP at mossy fiber inputs to CA3 pyramidal cells (Zalutsky and Nicoll, 1990), plasticity induction was NMDAR independent and did not require postsynaptic [Ca^2+^]_i_ rise, and expression was presynaptic. Remarkably, however, the outcome of plasticity was LTD rather than the LTP seen at pyramidal cells (Maccaferri et al., 1998). Further research (Lei and McBain, 2002; Galván et al., 2008, 2015) demonstrated that plasticity in CA3 interneurons was actually [Ca^2+^]_i_-dependent as it was blocked by fast calcium buffer BAPTA (in contrast to the slow buffer EGTA used in the Maccaferri et al., 1998 study), and revealed distinct sources for [Ca^2+^]_i_ rise and plasticity mechanisms in CA3 interneurons.

Mossy fiber synapses at str. lucidum interneurons contain calcium-permeable (CP) and calcium-impermeable (CI) AMPARs as well as NMDARs. The ratio of CP/CI-AMPAR expression covaried with the expression of NMDARs, forming a continuum from synapses with mostly CP-AMPARs and weaker and slower NMDAR-mediated components to synapses with mostly CI-AMPARs but a strong and fast NMDAR component (Bischofberger and Jonas, 2002; Lei and McBain, 2002). Calcium-dependent LTD induced by high-frequency stimulation was NMDAR-dependent and expressed postsynaptically at CI-AMPAR synapses but was NMDAR-independent and expressed presynaptically at CP-AMPAR synapses (Lei and McBain, 2004). Recent work suggests that induction of LTD at CP-AMPAR synapses involves release of BDNF from mossy fibers, which acts on postsynaptic TrkB receptors and triggers synthesis and release of endocannabinoids. Cannabinoids serve as a retrograde signal leading to reduction of glutamate release and, thus, presynaptic expression of LTD. When this signaling pathway was blocked or impaired, the proportion of LTD cases became smaller, but notably, LTP could also be observed (Pan et al., 2019).

At mossy fiber CI-AMPAR synapses onto CA3 str. lacunosum/moleculare interneurons, associative LTP could be induced by pairing high-frequency tetanization with depolarization (Galván et al., 2008). LTP was NMDAR-independent but required [Ca^2+^]_i_ rise *via* L-type voltage-gated calcium channels (VGCCs). Blockade of additional calcium sources, such as mGluR1α receptors or calcium release from intracellular stores *via* IP3 receptor or ryanodine receptor-mediated cascades, resulted in induction of LTD instead of LTP. Expression of both LTP and LTD involved presynaptic mechanisms (Galván et al., 2008). Because induction of LTP at mossy fiber synapses was not accompanied by significant changes at simultaneously tested associative-commissural inputs (95 ± 16% in *n* = 11 experiments), the authors concluded that LTP in str. lacunosum/moleculare interneurons was input-specific (Galván et al., 2008).

Synapses formed by axon collaterals of local pyramids onto CA3 str. radiatum and lacunosum/moleculare interneurons express CI-AMPARs, CP-AMPARs, and NMDARs, but the ratio of the CP/CI-AMPARs at a synapse did not correlate with the strength of NMDAR-mediated response component (Laezza and Dingledine, 2004). Induction of plasticity by afferent tetanization depended on the interaction between NMDARs, CP-AMPARs, and mGluR7s and the age of animals used for slice preparation (Laezza et al., 1999; Laezza and Dingledine, 2004; Galván et al., 2015). In slices from very young rat pups (P9–P12), the direction of plasticity at CP-AMPAR synapses was controlled by membrane potential during the tetanization. LTP was induced by tetanization at −30 mV or by pairing, but mostly LTD was observed after tetanization applied at 0 mV or −70 mV or with intracellular BAPTA. Blockade of NMDARs prevented induction of both LTP and LTD (Laezza and Dingledine, 2004). These results indicate that, at P9-P12, the bulk of calcium influx occurs *via* NMDARs and, when combined with the influx *via* CP-AMPARs, could provide [Ca^2+^]_i_ rise sufficient for triggering LTP. However, if influx *via* one or both sources is reduced or postsynaptic calcium is partially buffered, only the threshold for LTD induction is reached. In contrast to CP-AMPAR synapses, at P9–12, afferent tetanization at −30 mV did not induce plasticity in CI-AMPAR synapses.

In slices prepared from P10–P16 animals, plasticity at recurrent-collateral synapses also could be induced during NMDAR blockade: high-frequency stimulation induced LTD in CP-AMPAR-synapses, but LTP or no changes at CI-AMPA synapses. LTD at CP-AMPA synapses required [Ca^2+^]_i_ rise and activation of mGluR7 for the induction, and was expressed presynaptically (Laezza et al., 1999). The requirements for activation of distinct calcium sources for induction of plasticity further changed in older animals (P35 ± 5). With NMDARs unblocked, afferent tetanization induced LTD at recurrent-collateral synapses equipped with CP-AMPARs and LTP at CI-AMPAR synapses. LTP at CI-AMPAR synapses could be prevented by intracellular BAPTA or hyperpolarization to −100 mV during the tetanization. With NMDA receptors blocked, LTD was induced instead (Galván et al., 2015).

The requirement of NMDAR activation for LTP induction at recurrent-collateral synapses containing CI-AMPARs stands in contrast to the requirements for LTP induction at CI-AMPAR-synapses made by mossy fibers to the same neurons. LTP at mossy fibers was NMDAR-independent (Galván et al., 2015) but required calcium influx *via* L-type VGCCs (Galván et al., 2008). Distinct calcium sources activated distinct intracellular cascades: LTP at recurrent-collateral synapses involved CaMKII-signaling but not PKA-signaling while LTP at mossy fiber synapses was not impaired by the blockade of CaMKII-signaling but involved PKA-signaling (Galván et al., 2015).

To summarize, comparison of plasticity at three types of connections to CA3 interneurons (Figure 1, connections 5, 6, 7) supports the notion of the dependence of plasticity rules and mechanisms on the type of connection and on the pattern of expression of glutamate receptors at the tested synapses. Note, however, that differences in experimental conditions and plasticity induction protocols may have added to the diversity of results (see Table 1 and Box 3). For example, afferent tetanization of mossy fibers induced diverse forms of LTD in str. lucidum interneurons (Lei and McBain, 2002, 2004; Pelkey et al., 2005). The same tetanization but paired with depolarization of the postsynaptic cell could induce diverse forms of both LTP and LTD in str. radiatum and lacunosum/moleculare interneurons (Galván et al., 2008, 2015). Further research is needed to disentangle the role of variations in experimental conditions from the connection-specificity of plasticity mechanisms.

Age-dependence of plasticity mechanisms and requirements for specific sources mediating [Ca^2+^]_i_ rise for induction of plasticity in interneurons could be one further factor contributing to the variability of reported results. An emerging pattern is that, in very young animals, cooperative action of several sources is needed to rise [Ca^2+^]_i_ above the thresholds for plasticity induction. With maturation, individual sources become strong enough to provide [Ca^2+^]_i_ rise sufficient for the induction of plasticity. Because available data are sparse, this scenario is speculative.

#### Associative Plasticity of Mossy Fiber Inputs to DG Basket Cells Requires CP-AMPARs and mGluRs but Not NMDARs

Inputs from mossy fibers onto local interneurons in the dentate gyrus, PV-positive fast spiking basket cells, show bidirectional associative plasticity (Alle et al., 2001; Sambandan et al., 2010; Hainmüller et al., 2014). An “associative” induction protocol (burst frequency stimulation of mossy fibers paired with postsynaptic spikes) induced LTP in these cells while a “nonassociative” protocol (same burst frequency stimulation but paired with hyperpolarization preventing spikes) induced LTD (Alle et al., 2001; Hainmüller et al., 2014). Induction of LTP required [Ca^2+^]_i_ rise although it was attenuated only by high concentration of intracellular BAPTA, indicating a high capacity of intrinsic calcium buffers in these cells (Alle et al., 2001). LTP was NMDAR-independent, but required activation of CP-AMPARs, which are abundant at mossy fiber synapses on dentate gyrus interneurons (Sambandan et al., 2010). LTP induction also required activation of group I metabotropic glutamate receptors and PKC (Alle et al., 2001; Hainmüller et al., 2014). In the presence of mGluR1/5 blockers in the bath, associative burst-frequency stimulation induced LTD instead of LTP (Hainmüller et al., 2014). Expression of LTP and LTD involved presynaptic mechanisms as indicated by changes of the failure rate, paired-pulse ratio, and coefficient of variation (Alle et al., 2001).

### Interim Summary: Plasticity in the Hippocampus and Dentate Gyrus Interneurons

To summarize, research into plasticity in inhibitory neurons in the hippocampus showed that excitatory inputs to different types of inhibitory neurons express a multitude of forms and mechanisms of plasticity, including Hebbian and non-Hebbian-type plasticity at synapses activated during the induction (homosynaptic palsticity) as well as plastic changes at synapses that were not active during the induction (heterosynaptic plasticity; see below for detailed discussion).

Notably, the rules of induction and mechanisms of plasticity can be connection-type specific and determined by the properties and identity of both presynaptic fibers and postsynaptic cells. In the illustrative case of mossy fibers, canonical presynaptic LTP in CA3 pyramidal cells is purely presynaptic with the induction independent of postsynaptic calcium (Zalutsky and Nicoll, 1990). In contrast, at mossy fiber synapses formed on diverse types of interneurons, both LTP and LTD, with pre- or postsynaptic mechanisms of expression could be induced. Moreover, induction of plasticity invariably required postsynaptic rise of calcium (Laezza et al., 1999; Alle et al., 2001; Lei and McBain, 2002; Galván et al., 2008; Hainmüller et al., 2014), whereby the source of [Ca^2+^]_i_ rise and intracellular cascades leading to long-term plastic changes could be interneuron specific. For example, in dentate gyrus interneurons, LTP depends on CP-AMPARs (Sambandan et al., 2010), in interneurons from CA3 str. lacunosum/moleculare LTP depends on activation of L-type calcium channels and mGluR1-alpha (Galván et al., 2008), and in interneurons from CA3 str. lucidum, two different forms of LTD are induced depending on whether the tested synapses are equipped with a higher proportion of CP-AMPARs or with a stronger NMDAR-mediated response component (Bischofberger and Jonas, 2002; Lei and McBain, 2002, 2004). That latter example shows that plasticity mechanisms may be distinct even at synapses made at the same interneuron type by presynaptic fibers originating from the same source. One important consequence of the diversity of rules and mechanisms of plasticity is that the same pattern of activity may lead to different outcomes and even opposite-sign changes; e.g., in CA3 str. lacumosum/moleculare interneurons, tetanization of mossy fibers paired with postsynaptic depolarization leads to LTP at synapses equipped with CI-AMPARs but to no changes or LTD at CP-AMPAR synapses (Galván et al., 2008).

### Plasticity of Excitatory Inputs to Inhibitory Interneurons in the Neocortex

The diverse forms of connection-specific plasticity observed in hippocampal interneurons provide a framework for interpretation of sparse data on plasticity of excitatory inputs to inhibitory interneurons of the neocortex. Neocortical networks add several layers of complexity to research into plasticity in inhibitory interneurons due to an even higher diversity of interneuron types than in the hippocampus (Markram et al., 2004; Ascoli et al., 2008; Gentet, 2012; Battaglia et al., 2013; Druckmann et al., 2013; Jiang et al., 2015; Tremblay et al., 2016), the area-specificity of function and circuitry (e.g., in somatosensory, visual, prefrontal areas) and high heterogeneity of connectivity within each area, which essentially requires recording from connected pairs of neurons or using other means of identification of stimulated presynaptic fibers for obtaining data that are clearly connection-specific. Therefore, for neocortical interneurons, plasticity rules often can be related only to the properties of the postsynaptic cells.

In somatosensory cortex slices from young rats (P13–16), different plasticity rules were found at connections made by L2/3 pyramids onto either low-threshold spiking (LTS) or FS cells (Lu et al., 2007). In connections to LTS cells, conventional spike-timing-dependent plasticity (STDP) was observed after repetitive pairing of bursts of presynaptic and postsynaptic spikes. Pre-before-post pairing induced LTP, and pre-after-post pairing induced LTD at short intervals (8 ms). Both LTP and LTD were NMDA-dependent and not sensitive to mGluR blockade. In contrast, in connections from pyramidal cells to FS interneurons, only LTD was induced by either pre-post or post-pre pairing at short intervals. LTD in FS cells did not require NMDARs, but was prevented by the blockade of mGluRs (Lu et al., 2007). Plasticity windows were narrow in both LTS and FS cells; neither LTP nor LTD was induced after pairing with 25 ms or longer delays.

In mouse visual and somatosensory cortex, distinct mechanisms of plasticity have been reported for FS vs. non-FS cells. In FS cells from layer 2/3, theta-burst stimulation of input fibers from layer 4 induced LTP (Sarihi et al., 2008), and a conventional STDP protocol applied in the presence of agonists of α1 and β adrenergic receptors could induce LTP and LTD (Huang et al., 2013). LTP and LTD were not impaired by NMDAR blockade, but were prevented by the blockade of mGluR5. The theta-burst-induced LTP also could be prevented by blockers of the PLC-IP3 cascade and release from internal Ca^2+^ stores. In non-FS cells from L2/3 theta-burst stimulation of input fibers from layer 4 could also induce LTP but in only 6 out of 17 experiments and of a smaller magnitude than in FS cells (Sarihi et al., 2008). Like in FS cells, STDP could be induced in SOM-positive non-FS cells in the presence of agonists of α1 and β adrenergic receptors (Huang et al., 2013). Plasticity in non-FS interneurons was also NMDAR-independent.

**Figure 2:**
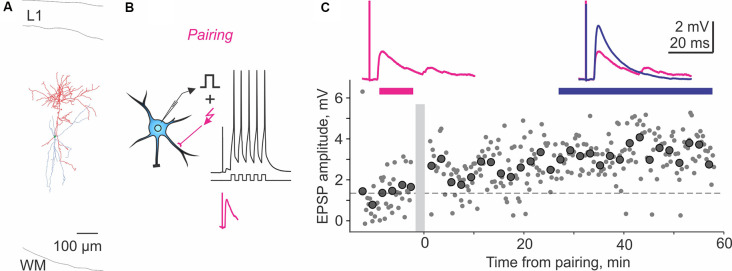
Homosynaptic long-term potentiation (LTP) induced by pairing procedure in a Martinotti cell from rat visual cortex. **(A)** Reconstruction of the Martinotti cell with dendrites in blue and axon in red. L1, layer 1; WM, white matter. **(B)** A scheme of a pre-before-post STDP pairing protocol, in which presynaptic stimulation was followed with a 10-ms delay, by a burst of postsynaptic spikes evoked by five depolarizing pulses at 100 Hz. Lower trace (magenta) shows zoom in of the EPSP without postsynaptic spikes. The pairing procedure was repeated 30 times. **(C)** LTP induced in the paired input. Response amplitudes (small symbols show individual responses; large symbols—averages over 2 min) plotted against time after the pairing (gray vertical bar). Horizontal dashed line shows average response amplitude during control period. Traces show averaged responses during the indicated periods before (magenta) and after (blue) the pairing. Data from the cell shown in panel **(A)**. Modified with permission from Chistiakova et al. (2019).

The following studies provide further evidence for plasticity in FS and non-FS interneurons but did not investigate its NMDA dependence. In the medial prefrontal cortex, theta-burst stimulation induced LTP in FS cells (Kerkhofs et al., 2018). In the visual cortex, repetitive pairing of presynaptic stimulation with bursts of postsynaptic spikes can induce long-term plasticity in both FS and non-FS cells from layers 2/3, 4, and 5 (Chistiakova et al., 2019). Pre-before-post pairing induced LTP in 5 out of 10 cells (Figure 2). Potentiation was significant also for the average of 10 paired inputs pooled together despite the fact that two of 10 cells expressed LTD. Notably, the pairing procedure also induced heterosynaptic LTP or LTD at inputs that were not stimulated during the pairing. However, because LTP and LTD at these inputs were about balanced, the average of all heterosynaptic inputs was not significantly different from control (Chistiakova et al., 2019). In the visual cortex, a form of age-dependent LTP induced by a mostly postsynaptic protocol has been described at unitary connections from star pyramids to layer 4 FS cells (Lefort et al., 2013). In reciprocally connected star pyramid–FS cell pairs, depolarization-induced bursts of high-frequency spikes in the FS neurons were combined with subthreshold depolarization of star pyramids. During this protocol, postsynaptic FS cells fired vigorously (~200 spikes) while presynaptic star pyramids may generate 1–2 occasional spikes. This protocol induced robust LTP in FS cells from P22–23 animals but not in younger animals.

The only form of plasticity in interneurons reported so far that did not require the rise of postsynaptic [Ca^2+^]_i_ was described in SST-expressing interneurons in mouse somatosensory cortex (Chen et al., 2009). This special form of LTP was induced by a very strong theta-burst stimulation (120–200 bursts of five stimuli at 100 Hz) and required cAMP-PKA signaling but was not impaired by intracellular BAPTA (30 mM), blockade of NMDAR, or L-type calcium channels. LTP expression involved presynaptic mechanisms. Note that, unlike in other studies of neocortical interneurons, experiments in this study were performed at room temperature (see Table 1), and the induction protocol was extremely strong. Conventional theta-burst stimulation (40–60 bursts) or afferent tetanization at 100 Hz did not induce this form of LTP (Chen et al., 2009).

Because of the diversity of investigated neocortical areas and heterogeneity of inhibitory neurons and connections, these data provide only a sparse and patchy picture. However, results are consistent with the interpretation suggested by research on hippocampal interneurons. All excitatory connections to inhibitory neurons studied so far could express long-term plasticity, including Hebbian-type bidirectional plasticity. In all but one report, induction of long-term plasticity required rise of postsynaptic [Ca^2+^]_i_. Both NMDAR-dependent and NMDAR-independent forms of plasticity are present in neocortical interneurons (Lu et al., 2007; Sarihi et al., 2008; Huang et al., 2013). Even sparse available data provide evidence for cell-type specific rules and mechanisms of plasticity in neocortical interneurons (e.g., PV-positive vs. SOM-positive cells, Huang et al., 2013), and the only study that compared properties of two clearly defined connections revealed that plasticity rules could be connection-specific (synapses made by pyramids onto FS vs. LTS cells, Lu et al., 2007).

### Modulation of Plasticity in Interneurons

Plasticity in inhibitory neurons is regulated by major neuromodulators, including acetylcholine, noradrenaline, adenosine, and glutamate (*via* mGluRs). Blockade of specific receptors to these neuromodulators could either prevent induction of plasticity altogether or mediate a switch between LTP and LTD.

Blockade of mGluR1/mGluR5s prevented induction of LTP in interneurons from CA1 str. oriens (Perez et al., 2001) and could switch the outcome of tetanization from LTP to LTD in interneurons from CA3 str. lacunosum/moleculare (Galván et al., 2008) and in the dentate gyrus (Hainmüller et al., 2014). In neocortical FS neurons from visual and somatosensory cortex blockade of mGluR5 prevented LTP induction by theta-burst stimulation (Sarihi et al., 2008) and prevented induction of LTP and LTD by an STDP protocol (Huang et al., 2013). Nonspecific blockade of mGluRs prevented paring-induced LTD at unitary connections from L2/3 pyramids to FS neurons in somatosensory cortex (Lu et al., 2007).

Cholinergic modulation of plasticity has been described in a subpopulation of SST-positive interneurons in CA1 str. oriens/alveus, which expresses calcium-permeable acetylcholine receptors (Jia et al., 2010; Griguoli et al., 2013). In these neurons, activation of nicotinic receptors was required for the induction of calcium-dependent, NMDAR-independent LTP by high-frequency tetanization paired with hyperpolarization. The two studies disagree on whether non-α7 nicotinic receptors (Jia et al., 2010) or α7 nicotinic receptors (Griguoli et al., 2013) mediated the calcium influx needed for LTP induction.

Requirement for activation of adrenergic receptors for the induction of bidirectional Hebbian-type plasticity by STDP protocol has been described in PV-positive FS and SOM-positive non-FS cells from the visual and somatosensory cortex (Huang et al., 2013). In both types of neurons, activation of β-adrenoreceptors was necessary for induction of LTP, and activation of α1-adrenoreceptors was necessary for induction of LTD. Without adrenergic agonists, no plasticity could be induced; in the presence of only β-adrenoreceptor agonists, only LTP and, in the presence of only α1 adrenoreceptor agonists, only LTD could be induced by both pre-before-post and pre-after-post pairing. With agonists of both α1 and β adrenergic receptors, canonical STDP was induced (Huang et al., 2013).

Modulation of plasticity by adenosine has been described for FS interneurons from prefrontal cortex (Kerkhofs et al., 2018). Theta-burst stimulation induced LTP in these interneurons in control conditions, but with adenosine A2 receptors blocked, the same stimulation induced LTD.

To summarize, major neuromodulatory systems are involved in regulation of plasticity in interneurons, and available data indicate that expression of distinct sets of neuromodulatory mechanisms may be among the factors that determine connection-specificity of plasticity rules.

## Calcium Sources and Intracellular Dynamics in Interneurons

### Common Aspects of Calcium Signaling in Interneurons

One common condition for induction of diverse forms of plasticity at excitatory inputs to interneurons is the requirement for postsynaptic [Ca^2+^]_i_ rise. With an exception of LTP induced by strong TBS in SST+/PV- non-FS interneurons in the mouse somatosensory cortex (which was not blocked by intracellular BAPTA; Chen et al., 2009), all other forms of plasticity in interneurons for which the effect of buffering of postsynaptic calcium was tested report that induction of plasticity was prevented (Cowan et al., 1998; Laezza et al., 1999; Wang and Kelly, 2001; Lei and McBain, 2002; Lamsa et al., 2005, 2007; Jia et al., 2010; Hainmüller et al., 2014; Nicholson and Kullmann, 2014) or attenuated (Alle et al., 2001).

#### Differential Calcium Thresholds for LTP and LTD in Interneurons

Results of research into how plasticity in interneurons is affected by manipulation of [Ca^2+^]_i_ rise, e.g., by modifications of induction protocols or partial block of calcium sources, are compatible with the idea of differential [Ca^2+^]_i_ thresholds for induction of LTP and LTD. This hypothesis has been initially proposed for pyramidal neurons (Bienenstock et al., 1982; Lisman, 1989, 2001). It postulates that [Ca^2+^]_i_ has to rise to a certain threshold to induce LTD and to a yet higher level to induce LTP. One prediction of this hypothesis is that, by reducing [Ca^2+^]_i_ rise produced by an “LTP-protocol,” e.g., by partial blockade of sources of calcium, it may induce LTD instead. Indeed, evidence from experiments in which diverse sources of [Ca^2+^]_i_ rise were manipulated supports this prediction. In CA3 str. radiatum interneurons, HFS applied at −30 mV induced LTP in control, but if [Ca^2+^]_i_ rise was reduced by intracellular BAPTA, LTD was induced (Laezza and Dingledine, 2004). In CA1 s.oriens/alveus interneurons, TBS paired with depolarization induced LTP if applied in control or with moderate reduction of [Ca^2+^]_i_ rise by blockade of either ERK or Srk or intracellular calcium release, but the same protocol induced LTD if [Ca^2+^]_i_ rise was reduced further by combined blockade of several of these sources (Topolnik et al., 2006). In mossy fiber inputs to dentate gyrus interneurons, associative burst-frequency stimulation induced LTP in control, but if calcium influx was reduced by the blockade of mGluRs1/5, LTD was induced instead (Hainmüller et al., 2014). Reduction of [Ca^2+^]_i_ rise could also result in a lower probability of LTP induction. In a subpopulation of SST-positive interneurons from CA1 str. oriens, HFS reliably induced LTP in control (*n* = 17 cells), but in only 6 out of 17 cells when [Ca^2+^]_i_ rise was reduced by blockade of calcium-permeable ACh receptors (Griguoli et al., 2013).

Notably, for some forms of plasticity in interneurons, the relation between the magnitude of [Ca^2+^]_i_ rise and induction of LTP or LTD could be different from that in pyramidal neurons, e.g., lacking the “LTD” window altogether (Le Roux et al., 2013) or even the inverse (whereby lower influx induces LTP and LTD occurring after higher rises). In FS interneurons from str. oriens of the CA1, subthreshold TBS leading to small amplitude Ca^2+^ transients induced LTP, but suprathreshold TBS leading to large supralinear Ca^2+^ signals in the dendrite-induced LTD (Camiré and Topolnik, 2014). Strong TBS could still induce LTP, if [Ca^2+^]_i_ rise is reduced and supralinear summation prevented by blocking calcium-dependent calcium release with CPA (Camiré and Topolnik, 2014). At mossy fiber synapses onto CA3 str. lucidum interneurons, high-frequency stimulation applied during the blockade of NMDARs induced LTD in 70 out of 84 cells (no changes in the remaining 14). However, when calcium influx was reduced by blockade of TrkB receptors or in TrkB knockout mice plastic outcomes shifted toward potentiation. LTP was induced in 11, and LTD in 12 out of 42 cells (Pan et al., 2019).

Thus, induction of LTP and LTD in interneurons can be related to the magnitude of [Ca^2+^]_i_ rise; however, in a cell-type specific way. The relationship of the outcome of plasticity to [Ca^2+^]_i_ rise can be similar or even opposite to the “canonical” dependence of thresholds for LTD and LTP induction in pyramidal neurons. One factor contributing to the observed diversity could be localization of calcium sensors of induction mechanisms relative to the sources of calcium influx.

#### Calcium Signals Are Local in Aspiny Dendrites of Interneurons

Synaptically evoked calcium signals in dendrites of interneurons can be local despite the absence of “restricting” morphological structures, such as spines. Several mechanisms, common for diverse types of interneurons, contribute to keeping calcium signals local in aspiny dendrites (Goldberg et al., 2003a; Kaiser et al., 2004; Goldberg and Yuste, 2005). Glutamate receptors mediating calcium influx in interneurons have rapid kinetics. This is true both for calcium-permeable AMPA receptors and also for NMDARs, which have faster kinetics in interneurons than in pyramidal cells (Bischofberger and Jonas, 2002; Lei and McBain, 2002, 2004). Interneurons have relatively thin dendrites and typically high buffer capacity for calcium (Matthews et al., 2013; Matthews and Dietrich, 2015) due to expression of diverse calcium buffers, such as calbindin, calretinin, or parvalbumin, which are hallmarks of diverse types of inhibitory neurons (e.g., Nissen et al., 2010; Gentet, 2012; Szabo et al., 2012; Tremblay et al., 2016; Pelkey et al., 2017). A combination of these factors—fast kinetics of channels mediating calcium entry, high buffering capacity, and thin dendrites—allows restriction of the spread of synaptic calcium signals (Box 2). Indeed, calcium imaging demonstrates that local synaptic activation in smooth dendrites produces microdomains of [Ca^2+^]_i_ rise restricted to one or few micrometer (Goldberg et al., 2003a; Kaiser et al., 2004; Rozsa et al., 2004). Thus, lack of spines does not prohibit localized calcium rise and signaling and, therefore, does not prevent induction of input-specific plasticity.

Note that calcium signals, mediated by ligand-gated mechanisms, are restricted to one or few μm around the activated synapse during responses to moderate levels of activity. Strong episodes of activity would expand [Ca^2+^]_i_ rise as more synapses distributed over larger portions of dendrites are engaged. In addition, strong activity may lead to spillover of transmitter and activation of extrasynaptic receptors in a broader region, which is, however, not clearly defined for physiological conditions. Spillover may engage ligand-gated mechanisms also at nearby dendrites, including dendrites of other cells within the spillover area, but still within a local region around the activated synapses.

Despite the common aspects of calcium signaling considered above, specific calcium sources, dynamics, and thresholds for induction of plasticity in interneurons are highly diverse and can be cell-type and connection-type specific. Below we first describe sources of calcium rise grouped into: (a) synaptic and other ligand-gated mechanisms; and (b) nonsynaptic, voltage-gated mechanisms, such as back-propagating action potentials and voltage-gated calcium channels, and then consider how the interaction of diverse mechanisms determines calcium dynamics in interneurons.

### Synaptic and Other Ligand-Gated Mechanisms Mediating Calcium Rise

Ligand-gated mechanisms contributing to the rise of [Ca^2+^]_i_ in interneurons include influx through NMDARs, calcium-permeable AMPARs, and calcium-permeable AChR channels and mechanisms coupled to mGluRs.

#### NMDAR and Calcium-Permeable AMPAR Channels

NMDAR channels represent a canonical source for the [Ca^2+^]_i_ rise that can trigger long-term plasticity in excitatory neurons (Bliss and Collingridge, 1993). In inhibitory neurons, expression of NMDARs, their contribution to the total calcium signal, and requirement for their activation for induction of plasticity is nonuniform. Typical ranges for NMDAR contribution to the calcium signal can be specific to distinct interneuron and connection types. In area CA1 of the hippocampus, quantitative immunogold labeling reveals that NMDARs, while consistently present at all spines on pyramidal cell dendrites, were found at low and variable density at dendrites of PV-positive interneurons with about 50% of dendrites lacking the label. Somata and dendritic shafts of SST-positive interneurons expressed highly variable density of NMDARs (Nyíri et al., 2003).

AMPARs in inhibitory interneurons can be calcium-permeable, depending on their subunit composition (Geiger et al., 1995; Jonas and Burnashev, 1995). AMPARs lacking the GluRB (GluR2) subunit have high permeability for calcium, fast kinetics, and are typically blocked by intracellular polyamines at positive potentials (Rozov and Burnashev, 1999). AMPARs containing edited GluRB subunit(s) have little calcium permeability, slow kinetics, and are not sensitive to polyamine block (see Box 2). The proportion of CP to CI AMPARs can differ systematically between cells of distinct types and can be different even at synapses originating from same presynaptic cells, e.g., in mossy fiber synapses to CA3 interneurons (Lei and McBain, 2002; Galván et al., 2008). In the neocortex, synapses made by pyramidal cells onto PV-positive basket cells express CP-AMPARs, but those at SST-positive Martinotti cells do not (Lalanne et al., 2016).

Although the identity of the postsynaptic cell is certainly the major determinant of the expression of postsynaptic receptors, their composition could be also connection-type specific, i.e., correlate with the source of presynaptic fibers that make synapses on the same postsynaptic neuron. Perisomatic inhibitory neurons in the dentate gyrus typically express more CP-AMPARs and less NMDARs at synapses received from mossy fibers, but more CI-AMPARs and more NMDARs at synapses made by the perforant path fibers (Sambandan et al., 2010; see Box 2). At mossy fiber synapses onto CA3 interneurons, expression of CP-AMPARs and NMDARs was inversely related: synapses with more CP-AMPARs contained less NMDARs, and vice versa, synapses with less CP-AMPARs but more CI-AMPARs contained more NMDARs (Lei and McBain, 2002). At recurrent collateral synapses made on these same cells by axon collaterals of local pyramids, no such correlation was found (Laezza et al., 1999; Laezza and Dingledine, 2004). In both mossy fiber and recurrent collateral connections to CA3 interneurons, more synapses were equipped with CI-AMPARs than with CP-AMPARs (Galván et al., 2008, 2015).

These results illustrating connection-specificity of the expression of CI-AMPARs, CP-AMPARs, and NMDARs, are in accordance with the NMDAR or CP-AMAPR-dependent mechanisms of plasticity revealed at respective synapses. Further support to the link between NMDAR and CP-AMPAR-mediated calcium influx on the one hand and specific forms of plasticity on the other comes from calcium imaging studies.

In the mouse visual cortex, in calretinin-positive irregular-spiking and adapting interneurons, NMDARs were the major source of calcium during synaptic stimulation. In these non-FS cells, blockade of NMDARs completely eliminated calcium signal in the dendrites or reduced it to <10% of control. In contrast, in PV-positive FS cells, blockade of NMDARs had variable effect on [Ca^2+^]_i_ rise, ranging between a complete block in 2 out of 17 cells, a negligible <10% reduction in two other, and intermediate reduction in the remaining 13 cells. The remaining calcium signal in FS cells could be blocked by AMPAR-antagonist DNQX or a selective CP-AMPAR blocker philanthotoxin and was, thus, mediated by CP-AMPARs (Goldberg et al., 2003c). Major contribution of NMDARs to [Ca^2+^]_i_ rise in all non-FS but in only few FS cells parallels results on NMDAR-dependent plasticity in non-FS neurons, and NMDAR-independent plasticity in FS neurons from the visual and somatosensory cortices, considered above (Lu et al., 2007; Sarihi et al., 2008; Huang et al., 2013).

In CA1 interneurons from str. oriens-alveus, including FS basket and bistratified neurons, synaptically evoked calcium signals were mediated predominantly by CP-AMPARs in 8 out of 14 cells, and predominantly by NMDARs in 6 out of 14 cells (Topolnik et al., 2005; Camiré and Topolnik, 2014). These results are paralleled by reports that, in most of these neurons “anti-Hebbian” LTP can be induced during NMDAR blockade (Lamsa et al., 2007), and either Hebbian or non-Hebbian LTP with NMDARs unblocked (Le Roux et al., 2013). In perisomatic interneurons in the dentate gyrus CP-AMPARs were the major source of calcium signal during burst-stimulation (Hainmüller et al., 2014), corresponding to the CP-AMPAR-dependent LTP in these cells (Sambandan et al., 2010).

An interesting aspect of calcium signaling *via* NMDARs and CP-AMPARs comes from a study of sparsely spiny FS interneurons. In these cells, a relative contribution of NMDARs and CP-AMPARs to the total calcium influx depends on whether the synapse is located on a spine or on a dendritic shaft. Both types of synapses may be equipped with both NMDARs and CP-AMPARs, but the proportion of NMDARs is higher at spines, and proportion of CP-AMPARs is higher at synapses made on dendritic shafts (Sancho and Bloodgood, 2018).

To summarize, cortical interneurons express plasticity forms that depend on calcium influx *via* NMDARs, CP-AMPARs, or both. Because of the different voltage dependence, calcium influx through CP-AMPAR and NMDAR channels is maximized in different ranges of the membrane potential (Box 2). Calcium influx through CP-AMPARs increases with hyperpolarization, and under physiological conditions is maximal at or below the resting potential, for example, when CP-AMPAR activation coincides with strong inhibition. Calcium influx through NMDARs is maximal at depolarized potentials around −50 mV to −30 mV, when the magnesium block is relieved, e.g., by strong excitation. This creates differential requirements for the induction of NMDAR or CP-AMPAR dependent plasticity (see section on plasticity and Table 1). Importantly, differential voltage-dependence of NMDARs and CP-AMPARs also expands the range of membrane potentials at which plasticity can be induced.

#### Metabotropic Glutamate Receptors (mGluRs)

Several types of metabotropic glutamate receptors (mGluRs) contribute to [Ca^2+^]_i_ rise and induction of plasticity in interneurons. In CA1 str. oriens/alveus interneurons, local puffs of agonists of group I or group I/II mGluRs could produce an increase of [Ca^2+^]_i_ in the dendrites (Gee et al., 2001; Topolnik et al., 2006). Calcium signals had either fast or slow kinetics and were mediated by distinct mechanisms. Fast signals were mediated by mGluR1α leading to activation of transient receptor potential (TRP) channels and release from internal calcium stores. Slow calcium signals were mediated by mGluR5 and exclusively by release from internal stores. Because mGluRs can be recruited by high-frequency or theta-burst stimulation (Topolnik et al., 2005, 2006), they could contribute to [Ca^2+^]_i_ rise needed for plasticity induction. Indeed, activation of mGluR1α was necessary for induction of LTP in str. oriens neurons (Perez et al., 2001; Topolnik et al., 2006; Griguoli et al., 2013). Activation of mGluR5 was necessary for induction of LTP in L2/3 interneurons from visual cortex (Sarihi et al., 2008), LTD at connections between L2/3 pyramids and FS interneurons in somatosensory cortex (Lu et al., 2007), and timing-dependent LTP and LTD by STDP protocol in PV-positive interneurons from visual and somatosensory cortices (Huang et al., 2013). mGluRs could also play a role of a switch from LTD to LTP, so that activation of mGluRs combined with other sources of calcium could produce [Ca^2+^]_i_ rise needed to induce LTP while, without mGluR activation, only the calcium threshold for LTD induction is reached. Indeed, at mossy fiber synapses onto perisomatic inhibitory neurons in the dentate gyrus (Hainmüller et al., 2014) and CA3 str. lacunosum/moleculare interneurons (Galván et al., 2008), LTP was induced in control conditions, but with group I mGluRs blocked, LTD was induced instead.

#### Calcium-Permeable Acetylcholine Receptors (CP-AChRs)

A subset of interneurons in CA1 str. oriens expresses calcium-permeable acetylcholine receptors (Jia et al., 2010; Griguoli et al., 2013). These cells were bistratified oriens-lacunosum/moleculare (O-LM) neurons and expressed SST and NPY but neither PV nor CB (Jia et al., 2010). With glutamatergic and GABA-ergic synaptic transmission blocked, cholinergic responses in these interneurons could be evoked by application of nicotine or synaptic stimulation (Jia et al., 2010; Griguoli et al., 2013). Rise of [Ca^2+^]_i_ in response to nicotine was mediated by both CP-AChRs and voltage-gated calcium channels (Jia et al., 2010). Calcium influx *via* CP-AChRs was necessary for the induction of NMDAR-independent “anti-Hebbian” LTP. Two studies diverge in identifying the specific subtype of nicotinic cholinergic receptors involved as non-α7 nAChRs (Jia et al., 2010) or α7 nAChRs (Griguoli et al., 2013), which could be due to the use of rats vs. mice for experiments.

#### Interim Summary: Ligand-Gated Mechanisms of Calcium Rise

To summarize, a variety of ligand-gated mechanisms mediate [Ca^2+^]_i_ rise in interneurons: NMDARs, calcium permeable AMPAR and AChRs channels, and metabotropic glutamate receptors. The set of mechanisms expressed at a synapse is naturally determined by the identity of the postsynaptic neurons (cell-specific); however, these sets also can be systematically different at synapses made at the same neuron by axons originating from different sources (connection-specific). At individual synapses, the contribution of diverse mechanisms to the total [Ca^2+^]_i_ rise vary markedly around the mean “connection-specific” values. Moreover, [Ca^2+^]_i_ rise at synapses of the same interneuron may be mediated by different combinations of ligand-gated calcium sources (Topolnik et al., 2005; Camiré and Topolnik, 2014; Sancho and Bloodgood, 2018).

### Nonsynaptic Mechanisms of Calcium Rise: Back-propagating Action Potentials and Voltage-Gated Calcium Channels (VGCCs)

Nonsynaptic mechanisms can mediate [Ca^2+^]_i_ rise that is not restricted to the dendrites contacted by activated synapses but can involve dendritic branches and, at maximum, the whole dendritic tree of the activated cell.

Interneurons, like pyramidal cells, express in their dendrites voltage-gated sodium and calcium channels, which support back-propagating action potentials (bAPs) and calcium influx (Martina et al., 2000; Kaiser et al., 2004). Dendritic calcium signals produced by bAPs have been reported for all interneurons studied so far, e.g., bitufted interneurons from L5 of visual cortex (Kaiser et al., 2004); multipolar FS PV-positive interneurons, bipolar irregular spiking CR-positive interneurons, and a heterogeneous group of adapting interneurons from L2/3 of visual cortex (Goldberg et al., 2003a, b; Sancho and Bloodgood, 2018); LTS SST-positive Martinotti cells from L5 of visual and somatosensory cortex (Goldberg et al., 2004); interneurons from str. radiatum of CA1 region in the hippocampus (Rozsa et al., 2004; Evstratova et al., 2011); CA1 oriens/alveus interneurons (Topolnik et al., 2009; Camiré and Topolnik, 2014); perisomatic interneurons from the dentate gyrus (Hainmüller et al., 2014). In interneurons, dendritic calcium signals are smaller and slower than in pyramidal cells. Back-propagation of APs into distal dendrites and related increase of [Ca^2+^]_i_ requires sodium channels and blockade of sodium channels with TTX typically restricts calcium signals to ~100 μm from the soma (Goldberg et al., 2003a; Kaiser et al., 2004; Evstratova et al., 2011). One remarkable exception here is active propagation of bursts of spikes into dendrites of LTS Martinotti cells from L5 of visual and somatosensory cortex. In burst mode, these cells can produce regenerative TTX-independent calcium spikes that propagate throughout the dendritic tree, and the amplitude of dendritic [Ca^2+^]_i_ rise can even increase with distance from the soma (Goldberg et al., 2004).

In all types of interneurons tested, bursts of spikes propagate into distant sites more effectively than single action potentials and evoke stronger calcium signals. The amplitudes of bAPs and related calcium signals in dendrites typically decay with distance, nonuniformly in neurons of diverse types. In CA1 oriens/alveus interneurons, simultaneous recordings from the soma and dendrites at distances up to ~100 μm revealed little decay of bAP amplitudes, which remained at 90% or higher of the somatic APs in most cells. The decay was similarly small for the first and the last AP in trains evoked by 100-ms pulses (Martina et al., 2000). In bitufted interneurons in L2/3 of the somatosensory cortex of rats, bAPs recorded at distances up to 50 μm from the soma had amplitudes above ~80% of the somatic (Kaiser et al., 2004). Calcium imaging demonstrated that bursts of bAPs can evoke in these cells dendritic [Ca^2+^]_i_ rises even at the maximal measured distance of ~400 μm. [Ca^2+^]_i_ rises in the distal dendrites, >200 μm from the soma, could have an amplitude comparable to that near the soma or be attenuated to ~20%–30% (Kaiser et al., 2004). In interneurons from mouse visual cortex, bAP-evoked calcium signals decayed faster with distance. In FS PV-positive interneurons with multipolar dendrites, irregular spiking CR-positive cells with bipolar morphology, and a heterogeneous group of interneurons with adapting firing pattern, the amplitude of calcium signals at >100 μm was about 30% of the amplitude close to the soma (Goldberg et al., 2003b). Backpropagation of APs and related calcium signals in these cells was restricted by activation of potassium channels. With potassium and sodium channels blocked, long depolarization pulses induced strong calcium signals that did not attenuate with distance, indicating that voltage-gated calcium channels in these cells could support calcium influx throughout the dendritic tree (Goldberg et al., 2003a). In several types of interneurons from mouse hippocampus CA1, including basket and Schaffer-collateral associated cells from str. radiatum and basket and bistratified cells from str. oriens/alveus, calcium signals induced by bursts of bAPs attenuated below detection level at ~150 μm from the soma (Evstratova et al., 2011; Topolnik, 2012; Camiré and Topolnik, 2014). An opposite situation with calcium signals increasing with distance from the soma has been reported for CA1 str. radiatum interneurons. The increment of bAP-evoked calcium signals measured in distal dendrites up to about 150–160 μm from the soma could be due to the small diameter of distal dendrites (Rozsa et al., 2004). Active propagation of TTX-resistant calcium spikes in LTS Martinotti cells from L5 of the neocortex (Goldberg et al., 2004), considered above, represents another example of a nondecremental spread of calcium signal over the whole dendritic tree.

Calcium influx during bAPs is primarily mediated by voltage-gated calcium channels and amplified by release from internal stores. Application of nonselective blockers or a cocktail of channel-type selective blockers of VGCCs reduced calcium signals to 10%–15% (Goldberg et al., 2003a; Rozsa et al., 2004; Topolnik et al., 2009; Evstratova et al., 2011). In interneurons of different types, distinct sets of VGCCs may mediate calcium signals. In CA1 str. radiatum, bAP-evoked calcium signals were mediated by a combination of L-, T-, and P/Q- type VGCCs in basket cells, but by L- and T-type, with negligible contribution of P/Q channels, in Schaffer collateral-associated cells (Evstratova et al., 2011).

### Interaction of Factors Determining Calcium Dynamics in Interneurons

Ultimately, dynamics of [Ca^2+^]_i_ in interneurons is determined by the interaction between multiple ligand-gated and voltage-gated sources of calcium influx described above as well as additional factors, such as calcium release from internal stores (e.g., Goldberg et al., 2003a; Topolnik et al., 2009; Evstratova et al., 2011; Camiré and Topolnik, 2014; Camiré et al., 2018) and internal calcium buffering and extrusion (Goldberg et al., 2003a; Rozsa et al., 2004; Evstratova et al., 2011; Matthews et al., 2013; Matthews and Dietrich, 2015; Chamberland et al., 2019).

Back-propagating APs, in addition to causing calcium influx *via* activation of VGCCs, can bidirectionally modify dendritic calcium signals produced by ligand-gated mechanisms. Back-propagating APs enhance NMDAR-mediated calcium signals in SST-positive and PV-positive interneurons from L2/3 of the visual cortex (Kaiser et al., 2004; Sancho and Bloodgood, 2018), similarly to the canonical mechanism of detection of coincident EPSPs and postsynaptic spikes in pyramidal neurons (Magee and Johnston, 1997; Markram et al., 1997). Also, mGluR mediated calcium signals in the dendrites of perisomatic inhibitory neurons in the dentate gyrus were enhanced by bAPs (Hainmüller et al., 2014). In contrast, [Ca^2+^]_i_ rise mediated by the CP-AMPARs is reduced by the spikes because of the decreasing driving force and eventual polyamine block at depolarized potentials (Rozov and Burnashev, 1999; Hainmüller et al., 2014; Sancho and Bloodgood, 2018). In FS interneurons from hippocampal CA1, strong stimulation of multiple presynaptic fibers can lead to supralinear summation of CP-AMPAR-mediated calcium signals due to calcium release from internal stores (Camiré and Topolnik, 2014; Camiré et al., 2018). Release from internal stores could also amplify calcium signals evoked by bAPs in several types of CA1 interneurons: CCK-positive basket cells and Schaffer collateral-associated cells from str. radiatum and interneurons from str. oriens/alveus (Topolnik et al., 2009; Evstratova et al., 2011).

One important factor that determines calcium dynamics in interneurons is high buffering capacity (Goldberg et al., 2003a; Rozsa et al., 2004; Evstratova et al., 2011). Expression of diverse calcium buffers, such as calbindin, calretinin, or parvalbumin, in neuron type-specific combinations (e.g., Nissen et al., 2010; Gentet, 2012; Szabo et al., 2012; Tremblay et al., 2016; Pelkey et al., 2017) results in marked differences between interneurons in calcium binding capacity (reviewed in Mattews et al., 2013; Matthews and Dietrich, 2015). The relation between calcium buffering capacity and calcium dynamics has been demonstrated in CA1 str. radiatum interneurons: calcium signals evoked by bAPs are larger and faster in basket cells with lower calcium buffering capacity than in Schaffer collateral-associated cells with higher capacity of calcium buffers (Evstratova et al., 2011). Type-specific differences in calcium buffering set distinct temporal and spatial restrictions on [Ca^2+^]_i_ rise and integration of calcium signals.

Sets of mechanisms mediating calcium influx while exhibiting a certain degree of specificity with respect to the type of interneurons and connections, may vary across cells and connections of the same type as discussed above. Moreover, synapses at the same neuron may express diverse sets of sources for [Ca^2+^]_i_ rises. Complementary sets of mechanisms mediating calcium influx at two dendritic locations of the same neuron had been clearly demonstrated in CA1 str. oriens interneurons. Calcium influx in response to glutamate puffs at one dendritic location was mediated almost exclusively by mGluRs with negligible contribution of NMDARs, AMPARs, and VGCCs while, at another location on the same cell, calcium responses were mediated by NMDARs, AMPARs, and VGCCs, and sequential blockade of these sources gradually reduced and eventually eliminated calcium responses (Topolnik et al., 2005). In sparsely spiny PV-positive interneurons from L2/3 of the visual cortex, synapses located on spines and on dendritic shafts both express CP-AMPARs and NMDARs, but the proportional contribution of NMDARs to calcium response in spines was about two times higher than at dendritic synapses (Sancho and Bloodgood, 2018).

To summarize, the requirement for [Ca^2+^]_i_ rise is one common condition for induction of long-term plasticity in inhibitory neurons. Multiple sources converge to contribute to the dynamics of intracellular calcium that ultimately determines whether and which intracellular mechanism(s) that may lead to long-term plasticity will be triggered. Manipulations that change the dynamics of intracellular calcium or the availability of intracellular cascades triggered by calcium rises may change the outcome of plasticity induction, e.g., between LTP and LTD.

## Heterosynaptic Plasticity of Excitatory Inputs to Inhibitory Neurons

Nonsynaptic mechanisms can produce [Ca^2+^]_i_ rise to the thresholds necessary to induce long-term plasticity not only at activated synapses, but also at nonactivated synapses, leading to heterosynaptic plasticity. By definition, heterosynaptic plasticity refers to changes at synapses that were not presynaptically activated during the induction protocol (Box 1). Initial studies of heterosynaptic plasticity in interneurons were motivated by the fact that interneurons have no or few spines on their dendrites with a majority of synapses made on dendritic shafts. Because spines restrict diffusion of molecules and ions, including calcium, the idea behind these experiments was that spread of intracellular calcium from active synapses along aspiny dendrites would facilitate induction of heterosynaptic plasticity at other, nonactivated synapses (McMahon and Kauer, 1997; Cowan et al., 1998). Indeed, in CA1 interneurons in the hippocampus, afferent tetanization induced LTD that could “spread” to nonactivated synapses (McMahon and Kauer, 1997) or induce plastic changes that lack input-specificity with LTP, LTD, or no changes occurring in both homosynaptic and heterosynaptic pathways in all possible combinations (Cowan et al., 1998). While the initial premise for lack of input specificity appeared to be wrong (aspiny dendrites do possess mechanisms that keep synaptically evoked [Ca^2+^]_i_ rises local; Goldberg et al., 2003a; Kaiser et al., 2004; Goldberg and Yuste, 2005), the above studies provide clear experimental evidence for heterosynaptic plasticity in hippocampal interneurons.

A large volume of subsequent work on interneurons from the hippocampus and neocortex reported only input-specific plasticity restricted to the synapses activated during the induction but no heterosynaptic changes (e.g., Lamsa et al., 2005, 2007; Pelkey et al., 2005; Galván et al., 2008, 2015; Sambandan et al., 2010; Huang et al., 2013; Le Roux et al., 2013; Nicholson and Kullmann, 2014). Note, however, that most of this research was aimed at in-depth analyses of specific forms of homosynaptic plasticity, and experimental conditions were optimized for the induction of these specific plasticity forms, e.g., induction protocols applied at hyperpolarized membrane potentials to maximize calcium influx *via* CP-AMPARs, and/or recordings were made with cesium-based intracellular solution and added sodium channel blocker QX-314 for better voltage control. Such experimental conditions, by impairing dendritic voltage-gated mechanisms and modifying calcium dynamics in the dendrites, might have impaired the induction of plasticity at heterosynaptic sites.

Calcium imaging shows that, in all interneurons studied so far, bAPs can propagate and evoke calcium signals in proximal dendrites, and in some types of interneurons reach distal parts of the dendritic tree (Rozsa et al., 2004) or even induce global dendritic calcium spikes, e.g., in LTS cells from L5 of the neocortex (Goldberg et al., 2004). Propagation of APs in a dendrite can be enhanced by depolarization produced by activation of synapses on that dendrite (for review see Goldberg and Yuste, 2005) or by downregulation of potassium channels that normally restrict backpropagation of APs (Goldberg et al., 2003b). Bursts of bAPs activate VGCCs that are present throughout the dendritic tree (Goldberg et al., 2003b). [Ca^2+^]_i_ rise mediated by VGCCs can be further amplified by release from internal stores (Topolnik et al., 2009; Evstratova et al., 2011). Combined action of these nonsynaptic mechanisms may rise [Ca^2+^]_i_ to the threshold for induction of long-term plasticity also at heterosynaptic sites within a dendritic branch that is currently most active or over broader regions of the dendritic tree.

The above scenario predicts that long-term plasticity in inhibitory neurons could be induced by strong postsynaptic activity without presynaptic activation. Indeed, in regular firing interneurons from CA1 region of the hippocampus, LTP could be induced by trains of postsynaptic spikes (about 600 APs) evoked by depolarizing pulses without presynaptic activity (Nicholson and Kullmann, 2014, 2017). LTP induced by AP trains shared common mechanisms of induction and expression with CP-AMPAR-dependent, NMDAR-independent LTP induced by afferent tetanization. Induction of LTP by either protocol was impaired by specific blockers of T-type calcium channels, indicating that T-channels contributed significantly to calcium influx (Nicholson and Kullmann, 2017). Moreover, LTP induced by AP trains showed two-way occlusion with the tetanus-induced LTP. LTP induced by either afferent tetanus or AP trains was associated with a decrease of the paired-pulse ratio and an increase of frequency of spontaneous EPSPs, suggesting involvement of presynaptic mechanisms in LTP expression. Interestingly, despite sharing mechanisms of induction and expression, tetanus-induced LTP was input-specific implying the need for presynaptic activation for the induction while LTP induced by AP trains was clearly independent of presynaptic activity at test synapses. One possible explanation for this difference is that nonsynaptic mechanisms of [Ca^2+^]_i_ rise, VGCCs, and release from internal stores were activated by the AP-only protocol sufficiently strong to produce calcium levels necessary for triggering plasticity all over the dendritic tree. During afferent tetanization, the [Ca^2+^]_i_ threshold for LTP was reached only around the activated synapses due to cooperative action of synaptic and nonsynaptic mechanisms of calcium rise, thus leading to input-specific LTP.

**Figure 3:**
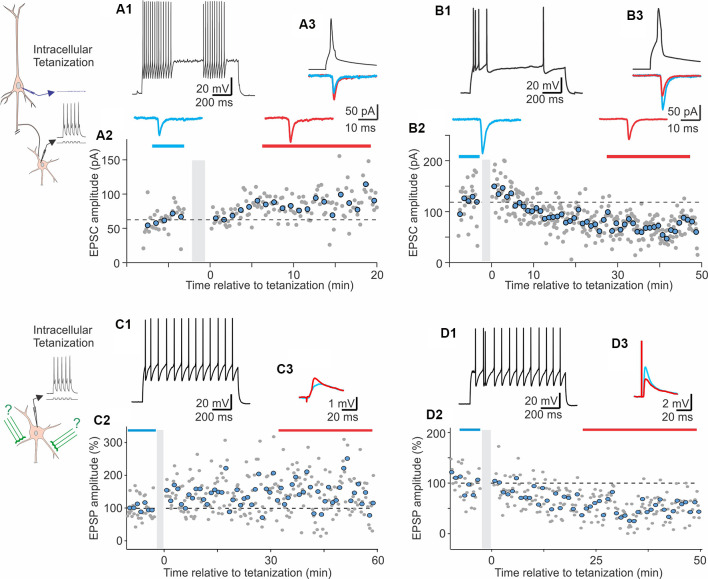
Heterosynaptic plasticity induced by intracellular tetanization in unitary connections from pyramidal neurons to inhibitory interneurons **(A1–A3, B1–B3)** and in pharmacologically isolated excitatory inputs to inhibitory neurons **(C1–C3, D1–D3)** in slices from rat visual cortex. Insets on the left show schemes of the intracellular tetanization experiment. Intracellular tetanization consisted of 30 bursts of five spikes evoked in the postsynaptic cell by short depolarizing pulses (5 ms, 100 Hz) without presynaptic stimulation. Note that, in experiments with unitary connections, absence of spikes in presynaptic pyramidal neurons was verified. In experiments with pharmacologically isolated EPSPs, 50 μm PTX was present in the extracellular medium throughout the recording. **(A1–A3)** LTP at unitary connection from a pyramidal cell to a fast-spiking (FS) inhibitory neuron from layer 3. **(A1)** Firing pattern of the postsynaptic FS neuron in response to a 1 s depolarizing pulse. **(A2)** Time course of unitary EPSC amplitude changes. Time of intracellular tetanization is indicated by vertical gray bar. Gray circles are individual amplitudes; larger blue circles are averages over 1 min. Horizontal dotted line shows mean response amplitude before tetanization. Averaged EPSCs are shown from the indicated periods before and after tetanization. **(A3)** Superimposed averaged EPSCs from **(A2)**, together with an average of presynaptic spikes. **(B1–B3)** Long-term depression (LTD) of unitary EPSCs in a non-FS neuron from layer 3. **(B1)** Firing pattern of the postsynaptic non-FS neuron. **(B2)** Time course of unitary EPSC amplitude changes and averaged responses before and after tetanization. Same conventions apply as in **(A2)**. **(B3)** Superimposed averaged EPSCs from **(B2)**, and an average of presynaptic spikes. **(C1–C3)** LTP of pharmacologically isolated EPSPs in FS neuron. **(C1)** Firing pattern of the FS neuron. **(C2)** Time course of EPSP amplitude changes. Time of intracellular tetanization is indicated by vertical gray bar. **(C3)** Superimposed averaged EPSPs from the periods before and after intracellular tetanization indicated on the time course. **(D1–D3)** LTD of pharmacologically isolated EPSPs in FS neuron. **(D1)** Firing pattern of the FS neuron. **(D2)** Time course of EPSP amplitude changes. **(D3)** Superimposed averaged EPSPs from the periods indicated on the time course (modified with permission from Chistiakova et al., 2019).

**Figure 4:**
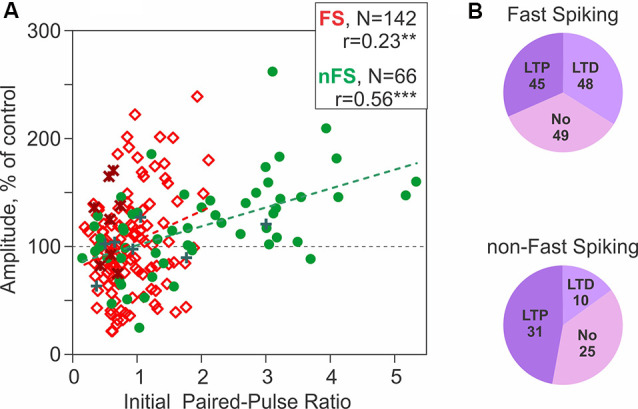
Distinct paired-pulse ratio and heterosynaptic plasticity in FS and non-FS cells from visual cortex. **(A)** Changes of EPSP amplitude after intracellular tetanization plotted against initial paired-pulse ratio for inputs to FS (red diamond symbols, *n* = 142) and non-FS (green circles, *n* = 66) neurons. In each group, initial PPR and EPSP amplitude changes were significantly correlated (**p < 0.01 and ***p < 0.001). Data for unitary responses from paired recordings are shown as dark red asterisks (FS, *n* = 8) and dark green crosses (non-FS, *n* = 8). Note that for excitatory inputs to FS cells PPR < 2 was typical while in non-FS neurons PPR > 2 were frequently observed. **(B)** Pie charts showing frequency of occurrence of LTP, LTD, and no changes after intracellular tetanization in FS and non-FS cells. Number of inputs contributing to each group is shown within the charts (modified with permission from Chistiakova et al., 2019).

Inhibitory neurons from the visual cortex also express heterosynaptic plasticity. A protocol of intracellular tetanization: bursts of postsynaptic spikes induced by depolarizing pulses without presynaptic stimulation, which induced long-term plasticity in excitatory neurons from visual and auditory cortex (Volgushev et al., 1997, 2016; Lee et al., 2012), also induced plasticity in inhibitory neurons (Chistiakova et al., 2019). Intracellular tetanization could induce LTP or LTD or lead to no synaptic changes in neurons of both FS and non-FS types. LTP or LTD could be induced in unitary connections between simultaneously recorded pairs of neurons with controlled absence of presynaptic spikes during intracellular tetanization (Figures 3A1–A3, B1–B3) and at excitatory synapses activated with extracellular electric stimulation (Figures 3C1–C3, D1–D3). Because intracellular tetanization is a purely postsynaptic protocol applied without presynaptic stimulation, any plastic changes occurred at nonactivated synapses and, thus, were heterosynaptic. The direction and magnitude of heterosynaptic changes were correlated with the initial paired-pulse ratio, an index of release that is inversely related to release probability (Voronin, 1993; Dobrunz and Stevens, 1997; Murthy et al., 1997). This correlation was significant for all studied inputs pooled together (*n* = 233 inputs) as well as for the subpopulations of inputs to identified FS neurons and identified non-FS cells (Figure 4). Thus, heterosynaptic changes in inhibitory neurons were weight-dependent: Inputs with initially high paired-pulse ratio (low release probability, “weak” inputs) tended to be potentiated while inputs with initially low paired-pulse ratio (high release probability, “strong” synapses) tended to depress or did not change after intracellular tetanization. LTP and LTD were balanced in FS neurons: an average of changes over all inputs to FS neurons did not show significant difference from control. In non-FS neurons, a higher proportion of inputs expressed LTP than LTD (Figure 4), and an average of all inputs to non-FS neurons showed significant potentiation. This difference might be due to a combination of (i) the correlation of plasticity with initial paired-pulse ratio and (ii) significantly higher paired-pulse facilitation ratios in the inputs to non-FS vs. FS neurons, resulting in an increased probability of LTP in non-FS cells. Notably, heterosynaptic plasticity in inhibitory neurons could also be induced by a conventional STDP pairing protocol (Chistiakova et al., 2019). Pre-before-post pairing of synaptic stimulation with bursts of depolarization-evoked postsynaptic spikes induced LTP in 5 out of 10 paired inputs (Figure 2), LTD in two, and did not lead to changes in the remaining three inputs. On average, paired inputs were significantly potentiated. Plastic changes were not restricted to the paired inputs: significant heterosynaptic LTP was observed in three, and LTD in 2 unpaired inputs out of 10. The average change in all unpaired inputs was not different from control. Thus, balanced heterosynaptic plasticity could be induced in inhibitory neurons by a conventional STDP protocol.

To summarize, there are form(s) of plasticity in interneurons that can be induced at synapses that were not active during the induction: heterosynaptic plasticity. Heterosynaptic plasticity can be induced by episodes of strong postsynaptic activity, evoked either purely postsynaptically by trains of depolarizing pulses (Nicholson and Kullmann, 2014, 2017; Chistiakova et al., 2019) or by conventional afferent tetanization (McMahon and Kauer, 1997; Cowan et al., 1998) or STDP pairing protocol (Chistiakova et al., 2019). Both LTP and LTD could be induced at heterosynaptic sites (Cowan et al., 1998; Chistiakova et al., 2019), whereby the direction of change is correlated with initial paired pulse ratio, suggesting weight-dependence of heterosynaptic plasticity (Chistiakova et al., 2019).

## How Diverse Forms of Plasticity Achieve Homeostasis of Excitatory Drive of Inhibitory Neurons

The majority of the input-specific plasticity discussed in this review can be classified as Hebbian-type associative plasticity. Associative plasticity is vital for adaptive fine-tuning of inhibitory systems to serve the multitude of their functions. However, Hebbian-type plasticity rules introduce an intrinsic positive feedback on synaptic weight changes, making synapses prone to runaway potentiation or depression and eventual saturation, and making neuronal activity prone to runaway activation or complete silencing. The need for homeostatic mechanisms to counteract these negative effects of Hebbian learning rules has been recognized since the earliest computational work on the subject (Von der Malsburg, 1973) and validated and specified in further work demonstrating that, to achieve both learning and stability of operation, neuronal networks need to be equipped with mechanisms of synaptic plasticity additional to Hebbain-type rules (e.g., Miller and MacKay, 1994; Miller, 1996; Oja, 1982; van Rossum et al., 2000; van Ooyen, 2001; Kempter et al., 2001; Wu and Yamaguchi, 2006; Morrison et al., 2008; Zenke et al., 2013). While these theoretical and computational studies had been focused on plasticity of excitatory connections between excitatory neurons, their results are not constrained by the transmitter identity of the output of the postsynaptic neuron. Inhibitory neurons driven by excitatory synapses equipped with Hebbian-type plasticity rules face the same problems: a tendency for runaway dynamics of synaptic weight changes and activity. Features of mechanism(s) counteracting these negative “side-effects” of Hebbian-type learning rules, established in theoretical work for excitatory neurons, are also relevant for mechanisms of homeostatic control of excitatory inputs to inhibitory neurons. The remarkable diversity of inhibitory neurons and plasticity mechanisms they express might impose additional constraints on the homeostatic mechanisms.

### Required Features of Mechanisms Balancing Excitatory Drive of Inhibitory Neurons

Homeostasis of synaptic weights should operate at several levels, keeping cells and synapses in their respective operational range. At the level of the whole cell, one function of homeostatic mechanism(s) is to preserve an overall synaptic drive and avoid excessive input changes, which may lead to runaway activation or complete silencing of a neuron. In theoretical and model simulation studies, homeostasis of total synaptic drive is typically achieved by normalization: after each iteration of learning and changes of the weights at a subset of synapses, the weights of all synapses are adjusted so that their total sum (or squared sum) remains constant (Von der Malsburg, 1973; Oja, 1982). While details of the normalization procedure may affect specifics of learning abilities of model neurons and networks, the normalization effectively maintains synaptic drive of a cell at a certain level and prevents runaway dynamics of activity (e.g., Miller and MacKay, 1994; Kempter et al., 2001; van Ooyen, 2001; Elliott and Shadbolt, 2002; Wu and Yamaguchi, 2006). However, maintaining the total weight of all synapses does not prevent saturation of individual weights or elimination of individual synapses. Indeed learning in models with normalization typically leads to a bimodal distribution of synaptic weights with the weights of the “winner” synapses at the maximum and weights of other synapses close to zero (e.g., Song et al., 2000; van Rossum et al., 2000; Gütig et al., 2003; Morrison et al., 2008). Synaptic weights of real neurons do not show such bimodal distributions, implying existence of additional mechanisms that prevent the saturation of weights of individual synapses. Thus, at the level of synapses, a function of homeostatic mechanisms is to prevent extreme changes of individual synaptic weights. This aspect of homeostasis is important for safeguarding synapses from elimination or saturation and keeping the weights in a range that allows for further learning and continued redistribution of weights to accommodate new memories (Volgushev et al., 2016).

One further general requirement for homeostatic mechanism(s) is the time scale of the induction of synaptic changes. Hebbian-type plasticity is induced within seconds or minutes, and to effectively counteract the tendency for runaway dynamics imposed by these fast plastic changes, homeostatic mechanism(s) should operate on a compatibly fast time scale (Wu and Yamaguchi, 2006; Zenke et al., 2013; Chistiakova et al., 2015; Zenke and Gerstner, 2017). Indeed, in most theoretical and simulation studies that use normalization to stabilize total synaptic drive, it is implemented directly into the equations for synaptic weight changes and, thus, operates on the exact same time scale as the associative plasticity (Von der Malsburg, 1973; Oja, 1982; Miller and MacKay, 1994; Miller, 1996). Research into the requirements for the time scale of homeostatic mechanisms showed that such mechanisms must induce “compensatory” plastic changes on the time scale that is same or similar to the time scale of Hebbian-type plasticity (Zenke et al., 2013; Zenke and Gerstner, 2017). One implication of this requirement is that mechanisms of “homeostatic synaptic scaling”, which induce plastic changes after many hours or days of dramatic alterations of activity level (Watt and Desai, 2010; Wenner, 2011; Turrigiano, 2012; Keck et al., 2017) and play a role during development or recovery after injury and deafferentation, cannot serve the homeostatic function for fast-scale Hebbian-type plasticity (for further discussion see Chistiakova et al., 2014, 2015; Zenke and Gerstner, 2017).

A common requirement for both homeostatic regulation and fine-tuning of inhibitory systems by associative plasticity is that synaptic weights could be changed in both directions. Synaptic weights that can only change in one direction will progressively saturate, lose dynamic range, and have no ability to support further plasticity. Indeed, both LTP and LTD were observed in many excitatory connections to inhibitory neurons considered in this review. In some connections, however, plasticity in one direction prevails, e.g., only LTP was reported so far at synapses made by axon collaterals of CA1 pyramids onto str. pyramidale interneurons mediating feedback inhibition (Lamsa et al., 2007; Le Roux et al., 2013) while, almost exclusively, LTD was observed at mossy fiber inputs to CA3 str. lucidum interneurons (Maccaferri et al., 1998; Lei and McBain, 2002, 2004; Pelkey et al., 2005). Because most of these studies were aimed at in-depth analysis of specific forms of plasticity and experimental conditions were optimized accordingly, further research is needed to determine conditions for bidirectional plasticity at the diverse types of excitatory synapses to inhibitory neurons.

The vast number of plasticity rules present in interneurons, along with their heterogeneous electrophysiological properties and diverse patterns of activity, set two further important constraints for homeostatic mechanism(s). To be successful, homeostatic mechanism(s) must be generic enough *to respond to a wide range of plasticity rules and mechanisms* and robust enough to serve this function under *a wide range of activity patterns of inhibitory neurons*, expressing these diverse forms of homosynaptic plasticity.

The diversity of plasticity in interneurons demonstrates the need for a generic homeostatic mechanism but also highlights a point of convergence of the requirements for plasticity induction: the rise of [Ca^2+^]_i_. It has been argued, in the broad context of synaptic plasticity at excitatory synapses, that an emphasis on [Ca^2+^]_i_ rise as the triggering mechanism of plasticity can offer improved explanatory value over a fixation on learning rules, such as STDP (Lisman and Spruston, 2005, 2010). This philosophy might more accurately capture the relevant point of convergence for a variety of plasticity rules. In interneurons, nearly all forms of associative homosynaptic plasticity reported so far are calcium-dependent (with only one exception discussed above; Chen et al., 2009). A homeostatic mechanism that is triggered by intracellular calcium would fulfill the requirement of being generic. Heterosynaptic plasticity is a calcium-dependent phenomena, whether the source of [Ca^2+^]_i_ rise is strong local activation and local spread to inactive synapses or more global influx through voltage gated channels activated by back-propogating action potentials and amplified by release from internal stores. Importantly, this form of plasticity can be initiated by any event that causes strong activation of a neuron, firing, and a rise of [Ca^2+^]_i_ to a sufficiently high level, meaning that it is capable of being engaged by almost any activity pattern that induced any of the diverse forms of homosynaptic plasticity discussed above.

The requirement for the homeostatic mechanism to be robust means that it must successfully prevent runaway synaptic dynamics across a broad range of input patterns and postsynaptic firing of electrophysiologically heterogeneous inhibitory neurons equipped with diverse plasticity mechanisms.

To summarize, an ideal candidate mechanism for counteracting tendency for runaway dynamics imposed by Hebbian-type learning rules on weight changes of excitatory synapses and activity in interneurons should fulfill the following requirements. It should be able to prevent both runaway dynamics of the total excitatory drive as well as extreme changes at individual synapses and divergence of the weights of all synapses to either a maximum or zero. It should operate on the time scale that is compatible with the time scale of the mechanisms of associative plasticity. It should be able to change synaptic weights in both directions. It should be generic, i.e., could be induced in conjunction with any of the diverse forms of Hebbian-type plasticity expressed in interneurons, and robust, i.e., serve the homeostatic function under a wide range of inputs and firing patterns of inhibitory neurons equipped with diverse plasticity mechanisms. At the same time, the homeostatic mechanism should not prevent associative learning and segregation of weights of synapses subject to different patterns of activity.

### Weight-Dependent Heterosynaptic Plasticity as a Candidate Mechanism for Homeostatic Regulation of Excitatory Drive to Inhibitory Neurons

The following observed properties of heterosynaptic plasticity at excitatory inputs to inhibitory neurons in the visual cortex (Chistiakova et al., 2019) allow it to fulfill the above requirements and serve the function of homeostatic regulation of synaptic weight changes.

Results from our recent study show that, in the visual cortex, both major types of interneurons, FS and non-FS cells, express weight-dependent heterosynaptic plasticity (Chistiakova et al., 2019). Thus, this phenomenon might be a general and robust feature of neocortical inhibitory neurons, which express a broad range of specific mechanisms of associative plasticity discussed above (e.g. Lu et al., 2007; Sarihi et al., 2008; Huang et al., 2013). Weight-dependent heterosynaptic plasticity is also present in pyramidal neurons from visual and auditory cortex (Volgushev et al., 2000, 2016; Lee et al., 2012; Chen et al., 2013), extending its generality as a widespread feature of neurons. It is calcium-dependent in pyramidal neurons (Lee et al., 2012) and might be triggered by [Ca^2+^]_i_ rise in inhibitory neurons as well. In interneurons, heterosynaptic plasticity could be induced by the same episodes of postsynaptic activity (bursts of spikes) as associative plasticity but at nonactive synapses. Because bursts of spikes induce [Ca^2+^]_i_ rise in any type of interneuron tested so far (Goldberg and Yuste, 2005; Topolnik and Camiré, 2019; see section on calcium above), and calcium rise is the trigger for associative plasticity, heterosynaptic plasticity might share this fundamental requirement. Additionally, heterosynaptic changes could be induced by the same protocols as homosynaptic associative plasticity; hence, both forms of plasticity operate on the same time scale. Thus, heterosynaptic plasticity fulfills the requirement of being generic because it is triggered by the same activity patterns that induce associative plasticity; it is capable of playing the role of homeostatic regulator of synaptic changes in a broad variety of neuron types; and further, it operates on the same time scale of associative plasticity.

In weight-dependent heterosynaptic plasticity, the direction and magnitude of synaptic changes depend on the initial strength of the synapse. Synapses that are initially weak will have a disposition to potentiate while synapses that are initially strong will be predisposed for depression. This weight dependence sets a background constraint on synaptic weight changes, which is able to control unstable dynamics regardless of the specifics of activity patterns that tend to induce it. Indeed, computer model simulations demonstrate that weight-dependent heterosynaptic plasticity can robustly prevent runaway dynamics of synaptic weights and runaway activity of model neurons subject to widely different patterns of activity and equipped with widely different plasticity rules (Chen et al., 2013; Volgushev et al., 2016; Bannon et al., 2017). Such universal homeostatic “brakes” on runaway dynamics allow learning networks to benefit from a broad variety of plasticity rules, STDP windows, and activity patterns while, at the same time, robustly maintaining stable regime of operation and keeping excitatory synapses in operating range allowing for new learning (Chistiakova et al., 2015, 2019).

Because of its weight dependence, heterosynaptic plasticity has a normalizing effect on synaptic weights, which prevents both excessive increases and excessive decreases of weights. An increase of the weight of a synapse increases its predisposition for depression and vice versa; a decrease of the weight will increase predisposition of the synapse for heterosynaptic potentiation. As a result, synaptic weights are driven away from extreme values toward an equilibrium point within the operational range. Importantly, this effect of heterosynaptic plasticity is different from the effect of a formal mathematical normalization. Mathematical normalization preserves total synaptic drive to a cell but does not prevent runaway potentiation or depression of individual synapses. Learning in such models typically leads to distribution of synaptic weights around two modes, at the maximal weight and around zero (Song et al., 2000; van Rossum et al., 2000; Gütig et al., 2003; Morrison et al., 2008). In contrast, in models with weight-dependent heterosynaptic plasticity, learning does not lead to runaway potentiation or depression of individual synapses. Rather, the weights of all synapses in such models remain within the operation range (Chen et al., 2013; Volgushev et al., 2016; Bannon et al., 2017). Thus, weight-dependent heterosynaptic plasticity can robustly prevent both runaway dynamics of total synaptic drive and activity of a neuron as well as excessive changes of weights of individual synapses.

Importantly, weight-dependent heterosynaptic plasticity does not prevent segregation of weights of synapses subject to distinct patterns of input activity, e.g., groups of inputs with different frequency or correlation of presynaptic firing (Chen et al., 2013; Volgushev et al., 2016). Rather, this mechanism enhances segregation of synaptic weights by introducing a background force on synaptic weight changes. Associative plasticity drives weights of active synapses toward either maximal or minimal values. Heterosynaptic plasticity, triggered by the same episodes of strong activity that induce homosynaptic associative plasticity, drives synaptic weights of all synapses, including those inactive, away from the extremes. In this scenario, changes of active vs. inactive synapses are driven by contrasting forces and have different target weights (Chen et al., 2013; Chistiakova et al., 2014, 2015; Volgushev et al., 2016).

Therefore, we conclude that weight-dependent heterosynaptic plasticity represents a strong candidate mechanism for homeostatic regulation of synaptic weights and balancing their changes during ongoing associative synaptic plasticity and learning in inhibitory neurons.

### Other Candidate Mechanisms for Balancing Plasticity at Excitatory Inputs to Inhibitory Neurons

Although the problem of balancing changes at excitatory synapses in interneurons during ongoing associative learning has received little attention so far, a large body of research into the same problem in excitatory neurons has suggested a number of solutions that could be applicable for inhibitory neurons too.

Several solutions aim at balancing bidirectional homosynaptic changes. Indeed, balance of synaptic changes in neuron models can be achieved by careful adjustment of plasticity windows in depression-biased STDP rules (Song et al., 2000; Kempter et al., 2001; Gütig et al., 2003; Babadi and Abbott, 2010). Such models can learn, e.g., input pattern discrimination, by driving synaptic weights to either a maximum or zero while maintaining stable mean firing rates (e.g., Song et al., 2000; van Rossum et al., 2000; Gütig et al., 2003; Morrison et al., 2008). A problem with this solution is that it requires a precise correspondence between the amplitude and duration of potentiation and depression windows in STDP rules on the one hand and frequency and pattern of the input activity on the other. A change of input activity would destabilize the neuron. In a population of neurons with different STDP rules, a common activity pattern could be destabilizing for some neurons. For the heterogeneous population of cortical interneurons expressing broad range of plasticity rules as discussed in this review, such a solution is too constrained to be plausible.

An elegant solution allowing a dynamic adjustment of plasticity rules in a neuron is a sliding threshold for LTP and LTD as proposed in the Bienenstock-Cooper-Munro model (Bienenstock et al., 1982). One suggested mechanism here is dependence of intracellular calcium homeostasis on the recent history of synaptic changes and activity (Yeung et al., 2004). Mechanisms for activity-dependent regulation of calcium housekeeping are reported at least for some inhibitory neurons. Intense synaptic activity could change calcium signals evoked by back-propagating action potentials in dendrites of CA1 interneurons (Topolnik et al., 2009; Evstratova et al., 2011). However, plasticity in some types of interneurons may differ from excitatory cells in its dependence on [Ca^2+^]_i_ rises (e.g., Camiré and Topolnik, 2014) or tetanization frequency (Le Roux et al., 2013) and may follow cell type-specific STDP rules (Lu et al., 2007). Therefore, further research is needed to understand how a mechanism employing sliding thresholds for LTP and LTD may operate in interneurons. Theoretical and computational analysis is needed to understand how specific plasticity rules and calcium thresholds in interneurons should be regulated to reconcile associative learning with stability of neuronal operation, and the existence of corresponding mechanisms in diverse types of inhibitory neurons requires experimental validation.

One further mechanism that can reduce effects of the positive feedback of Hebbian-type rules on synaptic weight changes is weight-dependence of associative plasticity. This mechanism has been suggested theoretically (Oja, 1982), and experimental results in excitatory neurons show that, while weak synapses can express strong potentiation, stronger synapses potentiate less (Bi and Poo, 1998; van Rossum et al., 2000; Hardingham et al., 2007). Weight-dependence slows down saturation of synaptic weights and helps to achieve stable activity level of model neurons (van Rossum et al., 2000; Gütig et al., 2003). It is logical to assume that associative plasticity at excitatory synapses to inhibitory neurons is weight-dependent too; however, details of such dependence in diverse types of inhibitory neurons need to be explored.

One common drawback of the above mechanisms using bidirectional homosynaptic plasticity to balance synaptic changes is that they require presynaptic activation of a synapse to adjust its weight but cannot affect inactive synapses. This reliance on an external factor, input activity at a synapse, limits the ability of these mechanisms to serve as cell-intrinsic regulators of synaptic homeostasis. We conclude that, while solutions based on homosynaptic plasticity may help balance synaptic changes (see Chistiakova et al., 2014, 2015; for review and further discussion), these mechanisms are neither robust nor generic and cannot universally accommodate the vast range of activity patterns and learning rules observed in interneurons.

Mechanisms that employ heterosynaptic changes do not have these limitations. A broadly defined group of mechanisms related to competition for resources could affect both presynaptically active as well as inactive synapses and may help to maintain an overall balance of synaptic weights (Frey and Morris, 1997, 1998; van Ooyen, 2001; Elliott and Shadbolt, 2002; Fonseca et al., 2004). Mechanisms from this group may be involved in mediating the weight-dependent heterosynaptic plasticity considered above. An interesting mechanism of local balancing of synaptic changes has been described in inhibitory neurons from basolateral amygdala. In these neurons, potentiated or depressed synapses are surrounded by changes of the opposite sign producing a locally balanced profile of synaptic changes (Royer and Paré, 2003). While neither competition for resources nor local balancing were studied in cortical inhibitory neurons so far, both mechanisms have potential to mediate a robust homeostatic regulation of excitatory inputs to cortical interneurons.

Finally, nonsynaptic mechanisms regulating intrinsic excitability could accompany synaptic plasticity in excitatory neurons (Bliss and Lomo, 1973; Daoudal et al., 2002; Zhang and Linden, 2003; Frick et al., 2004; Karmarkar and Buonomano, 2006; Fink and O’Dell, 2009; Sehgal et al., 2013). A whole neuron or just an activated dendritic branch may change excitability, thus affecting the constituent synapses. The effect of excitability changes may be either homeostatic, counteracting synaptic changes (Zhang and Linden, 2003; Karmarkar and Buonomano, 2006), or anti-homeostatic, enhancing synaptic changes (Frick et al., 2004; Fink and O’Dell, 2009). Note that the original study of Taube and Schwartzkroin (1987) did not find excitability changes in CA1 interneurons after tetanic stimulation. However, this issue requires further studies in other types of cortical interneurons, which express a remarkable heterogeneity of electrophysiological properties.

To summarize, we conclude that, among the mechanisms for homeostatic regulation of excitatory inputs to inhibitory neurons, considered above, weight-dependent heterosynaptic plasticity represents a strong candidate. It is a generic and robust mechanism that could serve the function of overall constraint of total synaptic weight (preventing extreme changes of synaptic drive and runaway activity) as well as the function of keeping weights of individual synapses in working range. Regardless, it is unlikely to be the only mechanism at work and additional mechanisms operating at the synaptic, cellular, and network levels might be involved in homeostatic regulation of activation of inhibitory neurons.

## Outlook and Open Questions: Why Is Plasticity in Interneurons Interesting?

Inhibitory interneurons exhibit unique morphology, electrophysiology, and patterns of protein expression, which clearly differentiate them from excitatory cells but, at the same time, are highly heterogeneous among themselves. This remarkable diversity of inhibitory neurons opens up an opportunity to address both cell-type and connection-specificity of plasticity rules as well as to distill basic rules common for all plastic synapses.

Diversity of distinct roles played by specific types of inhibitory interneurons in neuronal networks allows us to ask whether there are specific rules and mechanisms of plasticity that help to refine that circuit function. At the level of microcircuits, this could be studied, e.g., by comparison of plasticity in feed-forward vs. feedback inhibitory systems, or plasticity in inhibitory neurons targeting the dendrites and, thus, shaping input integration in pyramidal neurons vs. interneurons targeting the axon and the soma of pyramidal neurons and, thus, controlling their output. At the level of larger-scale cortical networks, relevant comparison(s) could be between plasticity in groups of inhibitory neurons serving distinct functions, e.g., mediating feature selectivity, shaping temporal patterns of activity and rhythms, or controlling and restricting spatial spread of activity. Progress of research that defines specific subpopulations and types of inhibitory neurons serving these and other specific functions opens up opportunities to address these kinds of questions.

Finally, achieving a better understanding of plasticity in inhibitory neurons has intrinsic value for the field of plasticity as a whole. One common motif of plasticity of excitatory inputs to inhibitory neurons discussed in this review is that individual synapses are typically equipped with mechanisms, such as distinct sources of [Ca^2+^]_i_ rise and intracellular machinery, which can support multiple forms of plasticity. How do these diverse mechanisms and forms of plasticity interact at one synapse? Most of the research has been aimed at disentangling the effects of specific mechanisms while their interaction received little attention so far. A further step toward understanding synaptic plasticity in inhibitory neurons would require knowledge of forms and mechanisms of plasticity that can be induced by natural patterns of activity, typical for each specific type of inhibitory neurons. The ultimate answer to this question would require studies during natural activity *in vivo* and should include modulation of plasticity rules by natural brain states.

## Author Contributions

NB, MC and MV designed and wrote the manuscript.

## Conflict of Interest

The authors declare that the research was conducted in the absence of any commercial or financial relationships that could be construed as a potential conflict of interest.
